# New insights for gynecological cancer therapies: from molecular mechanisms and clinical evidence to future directions

**DOI:** 10.1007/s10555-023-10113-2

**Published:** 2023-06-27

**Authors:** Chunxue Zhang, Yaru Sheng, Xiao Sun, Yudong Wang

**Affiliations:** 1 Department of Gynecologic Oncology, The International Peace Maternity and Child Health Hospital, School of Medicine, Shanghai Jiao Tong University, Shanghai, 200030 People’s Republic of China; 2 Shanghai Municipal Key Clinical Specialty, Female Tumor Reproductive Specialty, Shanghai, China; 3 Shanghai Key Laboratory of Embryo Original Disease, Shanghai, China

**Keywords:** Gynecological cancers, Novel agents, Targeted biomolecules, Clinical trials

## Abstract

Advanced and recurrent gynecological cancers lack effective treatment and have poor prognosis. Besides, there is urgent need for conservative treatment for fertility protection of young patients. Therefore, continued efforts are needed to further define underlying therapeutic targets and explore novel targeted strategies. Considerable advancements have been made with new insights into molecular mechanisms on cancer progression and breakthroughs in novel treatment strategies. Herein, we review the research that holds unique novelty and potential translational power to alter the current landscape of gynecological cancers and improve effective treatments. We outline the advent of promising therapies with their targeted biomolecules, including hormone receptor-targeted agents, inhibitors targeting epigenetic regulators, antiangiogenic agents, inhibitors of abnormal signaling pathways, poly (ADP-ribose) polymerase (PARP) inhibitors, agents targeting immune-suppressive regulators, and repurposed existing drugs. We particularly highlight clinical evidence and trace the ongoing clinical trials to investigate the translational value. Taken together, we conduct a thorough review on emerging agents for gynecological cancer treatment and further discuss their potential challenges and future opportunities.

## Background

Gynecological cancers, particularly endometrial, cervical, and ovarian cancers, have a significant impact on women’s health, with increasing incidence and mortality worldwide. Their symptoms and prognoses, epidemiologic and genetic risk factors, and individual responses to clinical therapy are all diverse. Additionally, the current challenges for management of gynecological cancers are the urgent need for conservative treatments for fertility preserve, especially in endometrial cancer (EC) due to the increasingly younger onset age, and patients in advanced-stage or recurrent condition have limited therapy options [1]; thus, a novel understanding of molecular and cellular biology is required for improving and personalizing drug development.

EC begins in the inner epithelial lining of the uterus (endometrium) [2]. Overweight and unopposed elevated estrogen levels are well-known risk factors for EC. Endometrial cancers are classified into four subtypes based on their molecular characteristics [3]: polymerase-epsilon (POLE) ultramutation, microsatellite instability (MSI) cluster, a copy-number low, and a copy-number high, each of which reveals a unique prognosis [3, 4]. Although the majority of cases are diagnosed after menopause and can be cured by hysterectomy, an increasing number of patients are younger than 40 years old, and most are nulliparous [5]. For patients in advanced stage and those who desire to protect future fertility or preserve their ovaries, there are fewer feasible treatments, which makes EC management challenging [6].

Cervical cancer (CC) is the fourth most common malignancy and the fourth main cause of cancer-related death among women, with an estimated 60,4000 new cases and 34,2000 deaths globally in 2020. The molecular pathogenesis of malignant CC is certainly influenced by exposure to human papillomavirus (HPV), which introduces related viral oncoproteins (E6, E7, and E5) and induces angiogenesis, DNA damage, dysfunction of the immune system, and epigenetic factors. Accordingly, HPV testing and HPV vaccines have been applied to screen and prevent CC. Recurrent and metastatic disease continues to be the leading cause of CC-related mortality, despite the fact that surgery, chemotherapy, and radiation therapy can cure approximately 90% of patients with early-stage cancer [7].

The 5-year survival rate for ovarian cancer (OC) has remained constant at 47% for the past 20 years, making it the deadliest gynecological malignancy. It is important to diagnose OC early, but only 15% of cases are diagnosed at an early stage. The standard treatments for OC include debulking surgery and platinum-based chemotherapy. The majority of patients will relapse within 3 years, despite the high response rate of the first-line treatment [8]. Upon first relapse, up to 25% of patients are platinum-resistant or platinum-refractory, and the response rates of single non-platinum agents (paclitaxel, docetaxel, pegylated liposomal doxorubicin (PLD), gemcitabine, and topotecan) and prognosis are disappointing [9, 10]. Thus, the deeper understanding of the underlying molecular mechanisms that contribute to cancer growth and chemoresistance is crucial to conduct new drugs and promising strategies for OC treatments [11].

**Fig. 1:**
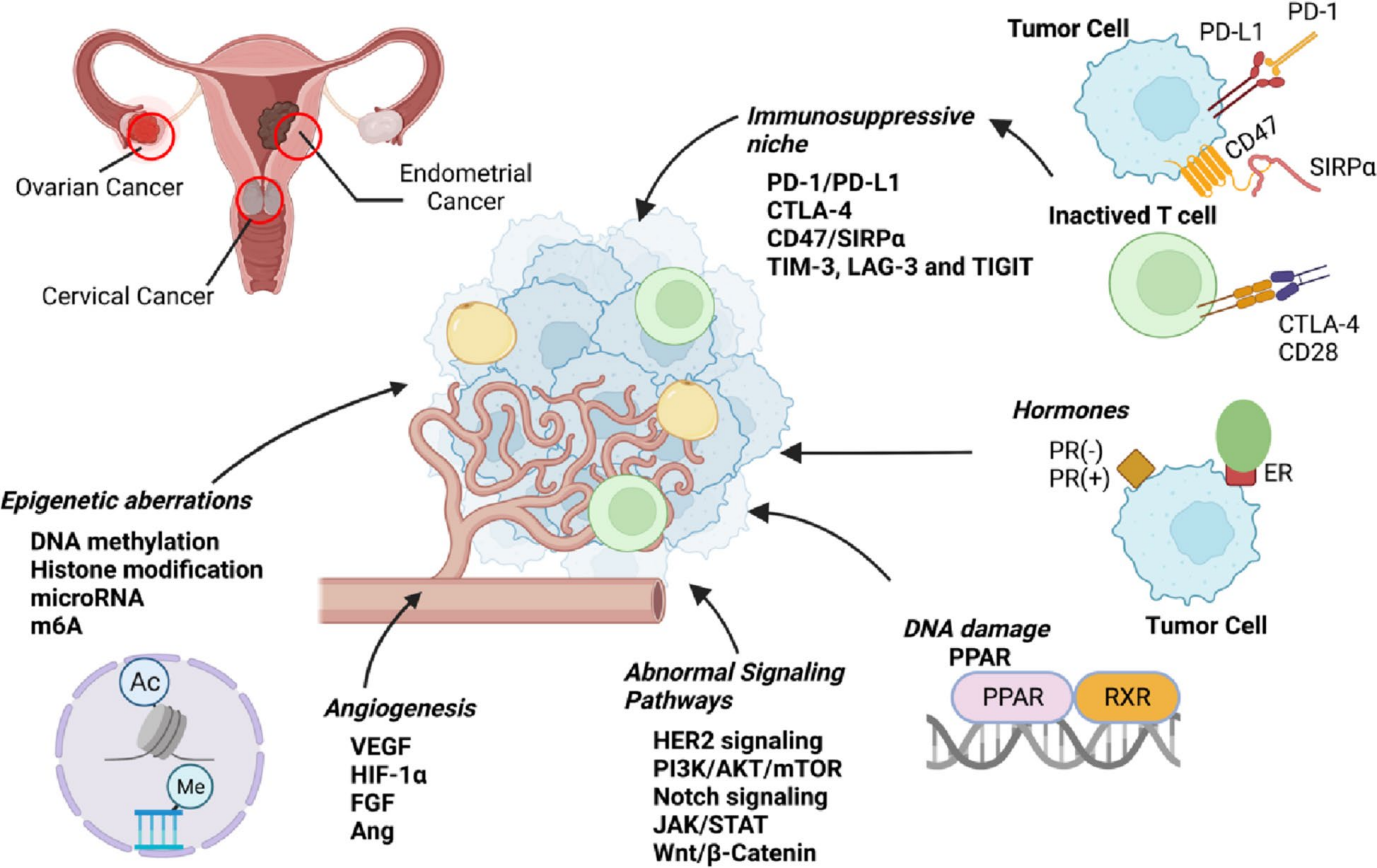
Schematic illustration of pathogenesis and therapeutic targets in gynecological cancers (created by BioRender.com)

In this paper, we summarize the outcomes of preclinical and clinical trials in endometrial, cervical, and ovarian cancers and review the Food and drug Administration (FDA)-approved drugs and the promising agents for gynecological cancer therapy (Fig. 1). We further describe their underlying molecular targets or mechanisms, as well as future directions.

## Methods

We thoroughly reviewed the literatures of promising agents and their clinical efficacies on gynecological cancers. To this end, published reviews, meta-analyses, clinical trials, and other observation studies were searched by PubMed. The information of the associated clinical trials which were completed or ongoing was collected from ClinicalTrials.gov. Based on FDA approval requirements, each approved targeted drug’s indications and references were searched on the website. The following terms were searched: gynecological cancers, ovarian cancer, cervical cancer, endometrial cancer, targeting agents, antiangiogenic agents, poly (ADP-ribose) polymerase (PARP) inhibitors, epigenetic inhibitors, immune checkpoint inhibitors, and each name of the targeted agents (e.g., “bevacizumab,” “pembrolizumab”).

## Hormone therapeutic strategies

The human endometrium thickens and renews itself to prepare for nourishing an embryo under the dynamic fluctuation of estrogen and progesterone [12–14]. However, hormone imbalance, either unopposed estrogen stimulation or insufficient progesterone conditions can result in endometrial pathologies, such as endometrial hyperplasia and endometrial cancer [15]. Obesity and excess exposure to hormone treatments are thought to be the main contributors of hormone imbalance in EC [16–18].

To counteract estrogen-induced endometrial proliferation, hormone therapies, including tamoxifen, levonorgestrel intrauterine device (IUD), and progestin (medroxyprogesterone acetate (MPA) and megestrol acetate (MA)), are prescribed for the adjuvant treatment of EC, the reversal of endometrial hyperplasia and the treatment of nulliparous women with low-grade EC [19]. By targeting specific receptors, progestin-mediated responses can impact the functions of numerous genes, such as *cyclin D1*, *Ets-1*, and *FOXO1*, and the activity of MMP proteases, which promote cell cycle arrest and apoptosis in EC cells [20]. The levonorgestrel IUD provides consistent localized progestin exposure, and it showed substantial activity in grade 1 endometrioid endometrial carcinoma in a prospective phase II trial, with minimal adverse effects and modest upfront progesterone resistance [21].

However, with the occurrence of receptor deficiency or drug resistance, the relapse rates of progesterone-treated EC are high [22]. It was reported that MPA treatment did not completely eradicate a carcinomatous lesion, which remained during and after a term pregnancy; therefore, these fertility-preserving options may be temporizing measures [23].

Several hormone therapeutic strategies have been investigated in recent years. The aromatase enzyme is responsible for converting androgen to estrogen and presents in 33–81% of ovarian tumor tissues [24]. Accordingly, aromatase inhibitors (letrozole and anastrozole) have received growing attention as therapeutic strategies against gynecological cancers [25, 26]. Letrozole exerts great antitumor effects and is well tolerated in patients with recurrent low-grade or borderline OC [27]. Letrozole combined with ribociclib (a cyclin kinase inhibitor) is safe and effective in patients with estrogen receptor (ER)-positive OC and EC, particularly in those with low-grade serous OC or EC, which represents a promising treatment option (NCT02657928) [28]. Recently, in a phase II study (NCT03675893), abemaciclib, a CDK4/6 inhibitor, in combination with letrozole exerted promising and durable antitumor efficacy in ER-positive EC patients with recurrent condition [29]. Anastrozole exhibits promising efficacy when combined with mTOR inhibitor vistusertib in hormone receptor-positive recurrent or metastatic EC patients (NCT02730923) [30].

Gonadotropin-releasing hormone (GnRH) signal transduction and intracellular actions are also involved for gynecological cancer therapy. GnRH is the central neuropeptide released from neurons in the hypothalamus and induces the synthesis and secretion of luteinizing hormone (LH) and follicle-stimulating hormone (FSH) from the anterior pituitary gland [31]. GnRH agonists (GnRH-as) are recommended in the treatment for OC and EC because of the critical role it plays in cell proliferation and metastasis. In EC, GnRH-as cause a reduction in cell proliferation by inhibiting epidermal growth factor receptor (EGF-R) signal transduction via extracellular signal-regulated kinase 1/2 (ERK1/2) or phosphoinositide 3-kinase (PI3K)/AKT pathway. GnRH also modulates apoptosis by activating nuclear factor-kappa B (NF-κB) or Fas ligand, and GnRH-as can activate the c-Jun N-terminal kinase (JNK)/activator protein-1 (AP-1) pathway, resulting in an increase of G0/1-phase cells and decreased DNA synthesis [32]. Furthermore, several studies have reported that patients treated with GnRH-as showed a significantly higher rate of implantation and clinical pregnancy than those treated with GnRH antagonists (GnRH-ants), while the mechanisms were unclear [33, 34].

Recently, androgen has been reported to be a new factor regulating squamous differentiation involved in the early progression of cervical intraepithelial neoplasia (CIN), suggesting a novel nonsurgical hormone-induced differentiation therapy could be used against CIN1 and CIN2 [35].

## Targeting epigenetic modifiers in gynecological cancers

**Fig. 2:**
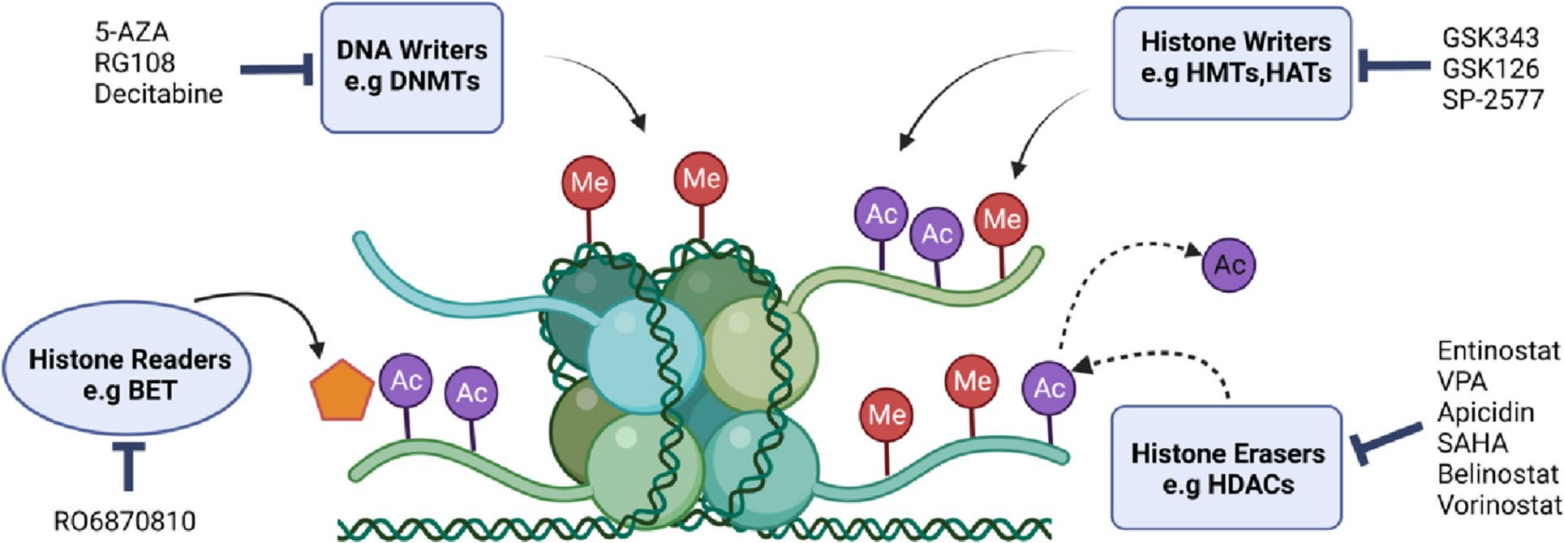
The conceptual diagram illustrating the functions and inhibitors of key epigenetic modifiers in gynecological cancers. DNA writers, such as DNMTs (DNA methyltransferases) add methyl groups (Me) and can be inhibited by 5-AZA (5-azacitidine), RG108, and decitabine. Histone writers, HMTs (histone methyltransferases) and HDMs (histone demethylases), add Me or acetyl groups (Ac) and can be inhibited by GSK343, GSK126, SP-2577. Histone erasers, HDACs (histone deacetylases), remove Ac and can be inhibited by approved drugs, including entinostat, VPA (valproic acid), apicidin, SAHA (suberoylanilide hydroxamic acid), belinostat and vorinostat. Histone readers such as the BET proteins can recognize acetylated lysine residues on histone tails, and they can be inhibited by R06870810 (created with BioRender.com)

Epigenetics is defined as heritable gene expression changes that do not alter the underlying DNA sequence. The epigenetic modifications mainly include histone methylation and acetylation, and DNA methylation [36]. In recent decades, the disturbances in epigenome resulting from epigenetic dysregulation have been proved to play a crucial role in the development and metastasis of a variety of cancers. Therefore, reprogramming the cancer-associated epigenome landscape is one of the most attractive targetable therapies in both reversing and treatments for a variety of malignancies [37]. Multiple inhibitors targeting epigenetic modifiers have been applied for cancer treatment for decades, predominantly in hematological disorders. At present, there are several candidate drugs targeting epigenetic-modified enzymes, typically DNA methyltransferase inhibitors (DNMTis) and histone deacetylase inhibitors (HDACis), entering clinical research, potentially providing new treatment approaches for patients with gynecologic cancers (Fig. 2) [38].

### DNA methylation events in gynecological cancers

DNA methyltransferases (DNMTs), such as DNMT1, DNMT3a, and DNMT3b, mediate DNA methylation in CpG islands [39], and aberrant DNA methylation is associated with endometrial tumorigenesis, induced by the change of gene transcription, including DNA repair factors, tumor suppressors and steroid receptors [40]. Interestingly, both type I and II EC exhibit aberrant DNA methylation profiles. In type I cancer cells, DNMT1 and DNMT3b were preferably upregulated, but downregulated in type II cancer cells [40]. In a retrospective study, elevated methylation of *hMLH1* and *O6-MGMT*, genes relating to DNA mismatch repair (MMR), were reported in atypical endometrial hyperplasia and continuously increased in tumor tissues, which seems to be an early event in EC carcinogenesis [41]. In addition, gene methylation, especially in *ADCYAP1* and *HAND2*, can be identified prior to the diagnosis of EC [42]. Hypermethylated *PCDHGB7* has been identified as a new cancer marker and is applicable in early screening for EC and CC [43]. Thus, abnormal DNA methylation could serve as a promising indicator for the detection of EC and CC.

In cervical carcinogenesis, alterations in DNA methylation pattern both affect the expression of persistent oncogenic HPV and disrupt cell cycle control, through which the epithelial host cells acquire immortal and malignant phenotype and further progress to invasive stage [44]. The fragile histidine triad, which acts as a negative cell growth regulator, is significantly downregulated in cervical neoplasia due to gene promoter hypermethylation [45, 46]. Transcription inhibition of the pro-apoptotic factor death-associated protein kinase (DAPK1) has also been reported in most cervical cancers [47, 48]. Similarly, *cyclin A1* (*CCNA1*) promoter hypermethylation, probably induced by the infection of HPV, is common in CC and the decreased gene expression is specific to the invasive phenotype [49, 50], suggesting a potential role of CCNA1 for early diagnosis of invasive CC. Hypermethylated *PCDHGB7* was also found in CC, which can be applied for early cancer screening [51].

A variety of genes were identified to be hypermethylated in the evolution of CC [52–60]. Ras association domain family 1 isoform A (RASSF1A) is a key regulator of apoptosis [61], and its gene promoter is uniquely hypermethylated in HPV-negative CC cell lines, but not in HPV-positive or primary cervical tumors [62]. In addition, *RASSF1A* promoter methylation was found in squamous cell carcinomas (10%), adenocarcinomas (20%), and adenosquamous carcinomas (45%), indicating that silenced RASSF1A may be involved in cervical adenocarcinoma progression [63, 64].

As for OC, aberrant DNA methylation is a contributing factor for tumor development and metastasis, chemotherapy resistance, and the survival of cancer stem cells [36]. It is possible to pinpoint specific methylated loci linked to poor progression-free survival (PFS) by comparing the level of aberrant methylation and the number of hypermethylated loci in OC, both of which are directly correlated with the progression and recurrence of ovarian tumors [65, 66]. Based on platinum-resistant DNA methylation signature, epigenetic therapies may reverse the transcriptional suppression that results in chemoresistance and restore sensitivity to platinum-based chemotherapeutics [67]. Homozygous methylation of *BRCA1* was viewed as a vigorous indicator of reaction to PARP inhibitors (PARPis) in an Ariel 2 clinical test of rucaparib. Furthermore, patterns of blood DNA methylation have been recently connected to prognosis of OC patients. Taken together, methylation markers in OC might be helpful for evaluation of therapeutic effects and recognition of chemoresistance-related pathways [68–71].

### Histone modification events in gynecological cancers

Histone methylation is regulated by histone methyltransferases (HMTs) and histone demethylases (HDMs). Methylation can occur both on the lysine residues (mono-, di-, or tri-methylation) and arginine residues (mono- or di-methylation) of histones. The dysregulation of these complicated methylation degrees was reported to be involved in gynecological cancers; thus, the modulators of histone-modifying enzymes have been in the forefront in gynecological cancer research because abnormal histone modification alters gene expression and may have adverse clinical effects [72].

Histone acetylation is regulated by the dynamic balance of histone acetyltransferases (HATs) and histone deacetylases (HDACs), which determines chromosome accessible or inaccessible to transcription factors and thus influences gene expression [73]. Two principal superfamilies of HATs have been recognized: the GNAT and MYST families, by which acetyl groups from lysine residues were added at the histone N-terminal tails [73]. The four classes of HDACs—class I (HDAC1, 2, 3, and 8), class II (HDAC4, 5, 6, 7, 9, and 10), class III (SIRT1, 2, 3, 4, 5, 6, and 7), and class IV (HDAC11)—are responsible for removing acetyl groups [74]. In addition, acetylation/deacetylation of histones influences the gene expression of nonhistone proteins, which are also involved in tumorigenesis, tumor progression, and metastasis [75, 76]. Thus, HDACis may be promising therapeutic targets for gynecological cancers.

HATs in EC are rarely reported, while numerous studies have shown that histone deacetylation causes tumor suppressor gene silencing and contributes to malignant transformation. Histone acetyltransferase MOF is known to regulate ER activity and maintain ER stability, which suppresses EC progression. The levels of HDACs (HDAC1, HDAC2, and HDAC3) are elevated in EC compared to normal endometrium, which have been reported to be related to a poor prognosis. On the contrary, higher SIRT1 level in EC is positively correlated with a better prognosis [77, 78].

For CC, elevated expressions of HDAC1 and HDAC2 were observed in both dysplasia and invasive carcinomas of cervical tissues. Overexpression of HDAC8 was reported in HeLa cells. Histone acetylation mainly affects crucial signaling pathways associated with CC progression by modulating the expression of key genes via acetylation/deacetylation. Loss of MGMT is linked to a decreased level of acetylated histones, which affects DNA repair in CC [79]. In HeLa cells, DICKKOPF-1 (DKK-1) is transcriptionally suppressed by histone deacetylation and inhibits the Wnt signaling pathway, which is crucial for HPV-infected cervical cells to undergo clonal proliferation at the early stage of malignancy [80]. It has been reported that the HPV E7 oncoprotein prevents HDACs from interacting with HIF-1 and results in HIF-1-dependent transcription [81].

The histone modification is essential for the tumorigenesis and progression in OC. Class I HDACs are elevated during ovarian carcinogenesis and are independent prognostic factors for malignant ovarian tumors. Compared with normal ovarian tissues, SIRT1 is significantly elevated in malignant ovarian tumors [82], and it was described to induce chemoresistance and correlate with poor prognosis in OC [83, 84].

### Interaction between DNA methylation and histone modification in gynecological cancers

Gynecological malignancies exhibit interaction between DNA methylation and the histone modification. In CC cell lines, aberrant histone modification and DNA methylation are responsible for silenced protein osteoprotegerin (OPG) [85], and retinoic acid receptor beta2 (RAR beta2) is epigenetically silenced either by DNA hypermethylation or repressive histone modification [86]. Liu et al. have demonstrated that HDAC1 and DNMT3a are linked to the suppression of octamer-binding transcription factor 4 (Oct4) in CC cells [87]. In EC, DICER1 regulates tumor invasion via histone acetylation and methylation [88]. These studies propose the possibility of combination treatment with DNMTis and HDACis for gynecological cancers.

### Emerging epigenetic targets for gynecological cancer treatment

#### Inhibitors targeting DNMTs, HMTs and HDMs, and HDACs in endometrial cancer

Epigenetic dysregulations, especially aberrant DNA methylation and histone modification, have been reported in the development and progression of EC, and several inhibitors targeting epigenetic regulators are considered to be effective against EC by a few preclinical studies [89]. Two emerging DNMTis have been investigated in EC cell lines. 5-azacytidine (5-AZA) suppressed EC cell proliferation through downregulating of cyclin D1 and β-catenin, while RG108 induced apoptosis of EC cells by demethylating the MMR gene *hMLH1* [90].

Inhibitors targeting HMTs and HDMs mainly include DOT1L inhibitors, EZH2 inhibitors, and LSD1 inhibitors, among which EZH2 inhibitors and LSD1 inhibitors exert therapeutic potential in EC. Studies demonstrated that EZH2 selective inhibitors significantly inhibited cell growth, of which GSK343 upregulated miR-361 and decreased Twist expression, and GSK126 induced apoptosis [91, 92]. A phase I clinical trial (NCT04611139) investigated the synergetic effect between LSD inhibitor (SP-2577) and pembrolizumab, an anti-programmed death 1 (PD-1) antibody on EC patients in 2020, however, it was withdrawn maybe due to severe toxicities.

Although more research has been done on histone acetylation than on histone methylation in EC, only one phase I clinical trial was initiated for EC treatment (NCT03018249) evaluating the therapeutic effect of combination with HDACi entinostat (MS-275) and hormonal therapy (MPA). Base on the knowledge that the expression level of progesterone receptor (PR) in the endometrium is positively related to MPA responsiveness, and epigenetically silenced PR resulted from histone modification could promote MPA resistance, the trial enrolled 22 patients in the MPA group and 20 in the entinostat/MPA group. Despite the fact that entinostat had no detectable impact on PR expression in this short period, the significantly decreased expression level of Ki-67 in the combination group compared to MPA group suggests entionstat exerts a synergetic effect with MPA in EC patients and this novel finding provides premise for progressing the combination strategy between entionstat and MPA towards a treatment trial [93].

#### Inhibitors targeting DNMTs and HDACs in cervical cancer

Hydralazine is a DNMTi that was well tolerated in clinical trial and plays a role in demethylating and reactivating several tumor suppressors in patients with CC [94]. Several HDACis have been reported to exert powerful anticancer effects in CC. Valproic acid (VPA), a potent HDACi, promotes apoptotic cell death by downregulation of Akt1, and it also hyperacetylates p53, which then increases p53 activity by preventing it from degradation by oncogenic HPV protein [95, 96]. In CC cells, the HDAC inhibitor Apicidin could preferentially downregulate DNM1 expression and induce repressive histone modifications by recruiting corepressor complex [97]. In HeLa cells, suberoylanilide hydroxamic acid (SAHA) synergistically causes apoptosis when treated with the proteasome inhibitor bortezomib through activation of caspase-3 and raising the bax/bcl-2 expression ratio [98].

#### Clinical trials of inhibitors targeting epigenetic modulators in ovarian cancer

DNMTis such as 5-AZA and decitabine, have been validated to be effective in regaining platinum sensitivity in platinum-resistant OC [99]. Decitabine performed better than 5-AZA to improve the responsiveness with a 35% objective response rate (ORR), a 10.2-month PFS, and the significant demethylation of tumor suppressor genes *MLH1*, *RASSF1A*, *HOXA10*, and *HOXA11* [100].

Belinostat, an HDACi, was once given to patients with platinum-resistant OC with no therapeutic results because of the termination due to serious side effects including neutropenia, thrombocytopenia, and vomiting [101]. Similarly, despite the partial response seen in clinical trial, vorinostat, a pan-HDACi, also caused severe hematological toxicities when treated with carboplatin or gemcitabine, resulting in the study’s cancelation [102].

Accordingly, clinical trials of epigenetic therapies in single-agent regimen have proved unsatisfactory for ovarian cancer treatment, so the focus of preclinical research has been shifted to the combination of various epigenetic medications. To improve anticancer therapy and overcome drug resistance for OC, epigenetic strategies have been initially combined with standard treatments in a few clinical trials.

The synergy efficiency of DNMTi and HDACi combination can be explained by the augmentation activity of HDACis in regulating chromatin accessibility and the recovery of abnormally silenced genes induced by DNMTis. In a xenograft model, the combination of decitabine and belinostat induced more platinum responsiveness in OC than either drug used alone [103].

It is generally acknowledged that combination therapy reduces drug toxicity in part because a low administered concentration has equivalent antitumor effects. However, in platinum-resistant epithelial ovarian cancer (EOC), the combination of 5-AZA, VPA, and carboplatin was extremely toxic, which resulted in the early termination of the study because 80% of participants experienced grade 3 or higher adverse effects, such as vomiting, neutropenia, and fatigue [104]. The severe toxicity profiles of 5-AZA and VPA may be because their targets span the epigenome, which emphasizes the necessity of more specific epigenetic modulators. Although the above clinical outcomes are less satisfactory, combined therapy is still a desirable treatment option, as many patients showed substantial and long-lasting clinical responses in non-small cell lung cancer when DNMTis and HDACis were combined [103].

Immunotherapy combined with DNMTis and/or HDACis enables the immune system to attack tumor cells unrestrainedly. In a syngeneic OC mouse model, combining decitabine and anti-CTLA-4 therapy dramatically inhibited tumor progression and extended survival when compared to either drug used alone [105]. Decitabine could stimulate the production of chemokines and attract CD8 and natural killer (NK) cells to the tumor microenvironment, which prolonged the cytotoxic lymphocyte response and then increased mouse survival. Epigenetic treatments combined with immune checkpoint inhibition have been tested in clinical trials based on the preclinical findings. Patients on a DNMTi combined with a HDACi mounted a potent and long-lasting response after receiving immune checkpoint therapy in non-small cell lung cancer, which supported the utility of triple combination therapy in solid tumors [106].

The combination therapy of entinostat (HDAC1/3 inhibitor) and avelumab, an antibody targeting programmed death ligand 1 (PD-L1), is now being tested in patients with chemo-resistant EOC, and the preliminary results are encouraging (NCT02915523). According to Odunsi et al., DNA methylation inhibits the expression of the cancer testis antigen NY-ESO-1. Combining a vaccination against this antigen with decitabine and doxorubicin induced a partial clinical response in six out of ten patients with recurrent EOC [107].

Other epigenetic modulators besides DNMTs and HDACs that are being studied in clinical trials include the histone lysine methyltransferase EZH2 and BET proteins which comprise BRD2, BRD3, BRD4, and BRDT and contain bromodomains that identify acetylated lysine residues on histone tails [108, 109]. BET inhibition suppresses the expression of MYC, an oncogene whose expression is favorably controlled by BRD4. BRD4 is typically overexpressed in OC and is linked to a poor prognosis. Cell cycle arrest is brought on by BET inhibition, which also prevents tumor growth [108]. BET inhibitors increase the DNA damage caused by PARP inhibition in cancer cells, mainly through lowering homologous recombination [110]. A phase Ib clinical trial combining RO6870810 with atezolizumab is also being carried out to evaluate the BET inhibitor and anti-PD-L1 therapy in advanced OC patients (NCT03292172).

### Other promising molecules involved in epigenetic regulation

#### MicroRNA-related epigenetic mechanisms in endometrial cancer

microRNAs (miRNAs) have drawn substantial interest in EC, from diagnostics and pathophysiology to therapeutics. miRNA-related epigenetic mechanisms can be summarized in three patterns: (1) miRNAs have the ability to directly bind to and silence target genes, serving as oncomiRNAs or tumor suppressors (such as miR-182 and miR-230); (2) CpG-rich regions in miRNA loci can be hypo- or hypermethylated, which leads to increased or decreased expression of these miRNAs, respectively (such as miR-34b and miR-129–2); and (3) miRNAs can increase/decrease the methylation of target genes (such as miR-30d and miR-191) [111]. In addition to their regulation of malignant cell phenotypes, miRNAs are also involved in chemosensitivity and angiogenesis in endometrioid EC [112, 113]. Through exosomal delivery, miRNAs (miRNA-21, miR-26a/b-5p) also mediate cellular communication between cancer cells and stromal or immune cells, which may be a potential mechanism underlying the construction of the immune microenvironment in EC progression [114, 115]. miR-26a-5p have also been demonstrated to contribute to lymph node metastasis, suggesting a specific target for EC therapies [116]. Due to their extensive implications in cancer progression, miRNA analysis has been explored as a promising factor in the management of patients with EC[117].

#### m^6^A modification in endometrial cancer

As the most common epigenetic modification of messenger RNA (mRNA), N6-methyladenosine (m^6^A) affects the pathogenesis of many diseases, including a wide range of cancers [118]. m6A modifications also intrinsically regulate tumor immunogenicity and modulate immune cells implicated in anti-tumor responses. Its dysregulation promotes cancer occurrence and development by driving aberrant transcription and translation programs, and immune cell responses in tumor cell are also affected by m6A alterations in the tumor microenvironment [36]. He Chuan et al. demonstrated that reduced m^6^A mRNA methylation is an oncogenic factor in EC [119]. Endometrial cancer cells proliferate and become more tumorigenic as a result of a mutation in *METTL14* (R298P), a crucial part of the methyltransferase complex, which activates the AKT pathway [119]. Demethylation of m^6^A modifications by *FTO* and *ALKBH5* promotes EC metastasis and invasion through the activation of Wnt and IGF1R signaling pathways, respectively [120, 121]. YTHDF2 is an m^6^A reader protein that promotes IRS1 mRNA degradation and inhibits cell proliferation and invasion by weakening IRS1/AKT signaling in EC [122]. These results suggest a protective role of m^6^A against EC progression. In liver cancer cells, YTHDF2 is positively correlated with the stem cell phenotype and cancer metastasis [123]. Insulin-like growth factor 2 mRNA-binding protein 1 (IGF2BP1), another “reader” of m^6^A sites in the 3′UTR of mRNA, enhances the mRNA stability of PEG10 and SOX2, thereby promoting cell cycle progression and tumor progression in EC [124, 125]. Thus, m^6^A modification may be a "double-edged sword" in endometrial tumorigenesis, and therapies targeting m^6^A should be thoroughly investigated before application.

## Angiogenesis and antiangiogenic agents

Angiogenesis is crucial for the development of tumors and the evolution of gynecological cancers in the female reproductive system. The understanding of vascularization in tumor growth has facilitated antiangiogenic therapy for gynecological cancers. Unfortunately, in a small subset of patients, antiangiogenic drugs have shown minimal clinical success, and despite initial benefit, many patients eventually acquire resistance to these drugs. Due to this inadequate efficacy, it is urgently necessary to identify new methods for controlling tumor vascularization and enhancing patient survival in ongoing preclinical investigations and clinical trials.

### The role of angiogenesis in gynecological cancers

#### Angiogenesis in endometrial cancer

As successful implantation and pregnancy depend on angiogenesis, the human endometrium shows stronger angiogenic potential than other female reproductive tract tissues. Due to an increase of proangiogenic and downregulation of antiangiogenic molecules, angiogenesis control is lost in CC. Furthermore, increased microvessel density (MVD) is positively correlated with aggressive phenotypes of tumor. The association between angiogenesis and estrogen signaling is a distinctive feature of EC, in addition to alterations of traditional angiogenic biomarkers in other tumors, including vascular endothelial growth factor (VEGF) and its receptors, hypoxia-inducible factor-1α (HIF-1α), fibroblast growth factor (FGF) and other angiogenic factors [126]. Previous studies have showed that estrogen promotes angiogenesis by inducing production of VEGF-A and the AKT, NF-κB or HIF-1 signaling are responsible for elevated VEGF levels. Through platelet-activating factor (PAF)-driven NF-κB activation and phospholipase A2 (PLA2) production, estrogen also promotes endometrial angiogenesis [126–128]. However, there is no evidence supporting the usage of estrogen signaling as a prognostic or predictive biomarker for EC.

#### Angiogenesis in cervical cancer

Angiogenesis is relevant in both premalignant cervical lesions and invasive CC. High vascularity is correlated with the poor prognosis of CC, and MVD is directly associated with VEGF expression [129]. HPV oncoproteins E6 and E7 have significant impacts on the angiogenesis of CC. E7 stimulates HIF-1 and inactivates the tumor suppressor protein retinoblastoma, and E6 promotes the degradation of p53, which are responsible for angiogenesis activation through the upregulation of VEGF [130, 131]. Furthermore, VEGF genetic polymorphisms influence cancer susceptibility and survival in early stage of CC through regulation of tumor angiogenesis [132–135].

#### Angiogenesis in ovarian cancer

EOCs are typically strongly vascularized, and the peritoneal vasculature is thick towards carcinomatosis, while the artery circulation is poor, which accelerate the development of edema and inflammation [136]. As the major subset of stromal cells in a number of human malignancies, cancer-associated fibroblasts (CAFs) can contribute to vascular stabilization in EOCs [137]. CAFs promote angiogenesis through activation of the tumor-derived proangiogenic growth factors, platelet-derived growth factor (PDGF), VEGF, FGF, and transforming growth factor-beta (TGF-β), and by secreting stromal cell-derived factor-1 (SDF-1), which draws endothelial progenitor cells to the tumor stroma [138].

Ovarian cancer stem cells (CSCs) also play a crucial role in the process of angiogenesis in malignancies [139, 140]. During hypoxia, the CSCs-derived angiogenesis serves as an alternative to sprouting angiogenesis, which arises from neighboring normal blood vessels and the interactions between ovarian CSCs. Besides, angiogenesis via VEGF, Wnt, Notch, and Sonic hedgehog signaling results in vascular cooperation, leading to metastasis of the tumor cells [141–143]. The anti-VEGF antibody bevacizumab significantly increased disease-free survival in both primary and recurrent OCs in clinical trials; hence drugs targeting CSCs that can enhance chemotherapy response and prevent recurrence will be the way forward [140].

### Clinical research of anti-angiogenic therapy

Bevacizumab monotherapy was investigated in a phase II clinical trial for patients with persistent or recurrent EOCs. The results showed a 21% clinical response rate, 4.7-month median PFS, and 17-month overall survival (OS), with favorable tolerability [144].

In addition, combination therapy of bevacizumab and chemotherapy was performed by numerous clinical trials. Patients who were treated with bevacizumab and chemotherapy (topotecan/paclitaxel or cisplatin/paclitaxel) had prolonged OS (17 versus 13.3 months), a longer PFS (8.2 versus 5.9 months), and a higher response rate (48% versus 36%) than those treated with chemotherapy alone, according to the Gynecologic Oncology Group (GOG)-240 phase III trial [145]. Accordingly, FDA approved bevacizumab plus chemotherapy as a combination therapy for metastatic and recurrent CC in 2014 [145, 146].

The addition of bevacizumab to carboplatin and paclitaxel chemotherapy followed by extended bevacizumab therapy (GOG-218 trial) in early diagnosed advanced EOC patients significantly prolonged the median PFS by 3.8 months compared to the chemotherapy plus placebo group. Meanwhile, this combination did not reduce patients’ quality of life [147]. Based on these findings, bevacizumab combined with platinum and paclitaxel has been approved by the European Medicine Agency (EMA) as a first-line chemotherapy regimen for OC.

The International Collaborative Ovarian Neoplasm (ICON) 7 trial showed a lower dose and a shorter maintenance period of bevacizumab in patients with OC. Compared with standard therapy (carboplatin and paclitaxel) group, patients assigned carboplatin-paclitaxel-bevacizumab regimen showed a better PFS, especially for those at high risk for progression (14.4 months vs. 18.1 months; *P* = 0.002). Additionally, patients with a poor prognosis had prolonged OS [148, 149]. This might be explained by the greater demand for angiogenesis in patients with high-risk progression, and the above combination strategy also prolonged the median PFS and OS in platinum-sensitive recurrent OC [150]. The combination of bevacizumab plus gemcitabine and carboplatin followed by bevacizumab maintenance therapy also led to significant improvement in PFS, ORR, and duration of response (DOR) in patients with platinum-sensitive recurrent EOC [151]. Based on these findings, both combination regimens were approved for platinum-sensitive recurrent OC. However, compared with carboplatin-paclitaxel, the treatment for advanced and recurrent EC with carboplatin-paclitaxel-bevacizumab did not significantly improve PFS in a randomized phase II trial (the MITO END-2 experiment) [152, 153].

Pazopanib and lapatinib are tyrosine kinase inhibitors (TKIs) that target VEGFR and PDGFR or EGFR and HER/neu, respectively. In a phase II clinical trial, pazopanib or lapatinib monotherapy or pazopanib-lapatinib combined regimen was given to patients with primary stage IVB or recurrent CC. When compared with lapatinib group, patients received pazopanib had significantly longer PFS (17.1 vs. 18.1 weeks; *P* = 0.013) and OS (39.1 vs. 50.7 weeks; *P* = 0.045). However, the combination therapy was low efficiency and more toxic than monotherapy [154]. As for the usage of pazopanib monotherapy in EC treatment, a case report showed that a 57-year-old patient with recurrent metastatic EC responded favorably to pazopanib monotherapy [155]. A phase III trial (OVAR-16) testing pazopanib maintenance therapy for OC patients whose disease did not progress during first-line chemotherapy showed a significant improvement in the median PFS, while there was no benefit in OS [156]. A phase II trial (MITO 11) enrolled patients with platinum-resistant or platinum-refractory advanced OC and found that PFS was greatly longer in the pazopanib plus paclitaxel group than that in paclitaxel group (6.35 months vs. 3.49 months; *P* = 0.0002), and that the adverse events, such as neutropenia, fatigue, leucopenia, and hypertension, were more common in the combination group [157].

Nintedanib is an oral triple angiokinase inhibitor of VEGFR, PDGFR, and EGFR. In newly diagnosed advanced OC patients, the phase 3 trial AGO-OVAR 12 investigated the combination therapy of nintedanib with the standard treatment (carboplatin and paclitaxel). The results showed that patients who received carboplatin-paclitaxel-nintedanib therapy had longer PFS than patients from the carboplatin-paclitaxel-placebo group [158].

Cediranib is an oral antiangiogenic VEGFR1/2/3 inhibitor. According to a phase 3 trial (ICON6), when combined with chemotherapy and continued as maintenance therapy, cediranib greatly increased PFS in recurrent platinum-sensitive OC patients, albeit with added toxic effects, which was similar with the PFS benefit of bevacizumab observed in the GOG-0213 and OCEANS trials [159]. Considering that TKIs are commonly multitarget inhibitors, the balance between increased toxicity and clinical benefits needs to be further investigated in future trials.

A single-arm clinical trial (AMG 386; IND#111,071) of 32 patients with persistent/recurrent EC assessed anti-angiopoietin therapy with trebananib, a peptibody that selectively neutralizes angiopoietin 1/2. The OS and PFS were 2.0 and 6.6 months, respectively. Eight patients exhibited stable progression, five had 6-month event-free survival, and one patient displayed a partial response. Unfortunately, trebananib monotherapy has no obvious efficiency [160].

## Therapeutic agents targeting abnormal signaling pathways

Tumor-intrinsic signaling pathways that drive cancer initiation and progression provide intriguing targets for antitumor therapies. Due in part to the upregulated expression of drug transporters and reprogramming of the resistant microenvironment, CSCs are responsible for metastasis, therapy resistance and cancer relapse [161, 162]. In EC, stemness and cell fate are tightly modulated by abnormal signaling pathways, such as Wnt/β-Catenin, Notch, and PI3K/AKT/mTOR signaling [163, 164]. Dysregulations of components in these cascades have also been identified in several OC subtypes [165], and the stemness of ovarian CSCs is directly regulated by PI3K/PTEN/AKT pathway, which causes enrichment and phenotype maintenance of CSCs, as well as promotes multidrug resistance [166]. Currently, in gynecological malignancies, several molecules have been targeted for promising therapeutic strategies, including HER2, PI3K/mTOR, and other signaling targets.

### HER2-targeted inhibitors

Targeting HER2 has been established as a therapeutic strategy for large subsets of breast cancer patients. Accordingly, trastuzumab, pertuzumab, lapatinib, neratinib and trastuzumab emtansine (T-DM1) have been approved for the treatment of HER2-positive breast cancers [167].

High gene amplification and/or protein expression of HER2 is also involved in EC, especially in serous carcinomas, which provides an opportunity for targeted therapy [168, 169]. Although no HER2-targeting agents have been approved for gynecological cancer treatment, a number of translational studies and clinical trials are in progress, which may alter the paradigm of treating gynecologic cancers that express HER2 [170].

Pertuzumab is a monoclonal antibody against HER-mediated signaling and tolerated in relapsed OC patients, with response rate (RR) of 4.3% [171]. For advanced, platinum-resistant EOC patients, the combination of pertuzumab and gemcitabine demonstrated a higher RR and a trend towards improved PFS[172]. However, pertuzumab did not substantially favor carboplatin-based chemotherapy with a comparable PFS in platinum-sensitive OC patients [173]. The randomized phase III clinical research demonstrated that the combination of pertuzumab with chemotherapy did not greatly increase PFS [174].

In a clinical trial evaluating the feasibility of trastuzumab in OC, the overall RR of 7.3% suggested a limited clinical value for single-agent monoclonal antibody therapy, which may be due to low objective response or low incidence of HER2 overexpression [175]. Single-agent trastuzumab therapy also demonstrated few activities against EC even with HER2 amplification or overexpression [176]. However, trastuzumab increased PFS and OS when combined with carboplatin/paclitaxel in patients with HER2/Neu-positive advanced or recurrent uterine-serous-carcinoma (USC) [177], and trastuzumab appears to be safe and exhibit a manageable toxicity profile in patients with HER2/Neu-positive USC, both in combination with chemotherapy and as a single-agent maintenance [178]. The combination therapy of trastuzumab and carboplatin-paclitaxel significantly prolonged PFS without unexpected safety signals in USC patients with HER2/neu overexpression [179]. Adavosertib is a specific and effective inhibitor of the WEE1 kinase. The monotherapy of adavosertib demonstrated durable and promising anti-tumor efficacy in USC (29.4% ORR) [180]. In addition to the direct cytotoxic effect in gynecological cancers, adavosertib also reversed trastuzumab resistance in HER2-positive cancers, which supports further clinical development [181, 182].

Currently, the therapeutics targeting HER2 face many challenges in CC [183]. Firstly, a truncated form of HER2, p95HER2, was reported to be highly expressed in high-grade EC, which led to trastuzumab resistance due to the lack of N-terminal trastuzumab binding. Secondly, abnormal activation of the PI3K pathway and Notch signaling are associated with the resistance of HER2 therapies [183, 184]. On the contrary, HER3 was increased upon PI3K pathway inhibition via mTOR, AKT, or direct PI3K blockage. Thirdly, the expression of EGFR (HER1, ERBB1) was also related to decreased sensitivity of trastuzumab [183].

### PI3K/AKT/mTOR signaling and targeted inhibitors

The PI3K/AKT/mTOR pathway is involved in the pathogenesis of gynecological cancers and in crosstalk with the estrogen receptor signals and RAS/RAF/MEK pathways [185]. Mechanisms underlying abnormal PI3K/AKT/mTOR pathway activation include amplification or mutation of PI3K/AKT, activation of growth factor receptors, loss function of tumor suppressor PTEN, and exposure to carcinogens [186]. Through a diverse set of downstream targets, the PI3K/AKT/mTOR pathway responds to several endogenous or exogenous stimuli to determine EC cell fate and AKT can be catalytically activated by the MLLT11-TRIL complex, which promotes cancer progression [187]. In EC, PI3K/AKT/mTOR inhibition is responsible for metformin-induced cell proliferation, while its activation promotes progestin resistance [188, 189]. In EOC, abnormal activation of AKT is closely linked with poor PFS and OS [190]. The stemness of OC is directly regulated by PI3K/PTEN/AKT pathway, which mediates stem cell enrichment and phenotype maintenance, as well as multidrug resistance [166], resulting in aberrant cell proliferation and epithelial-mesenchymal transition (EMT) [191].

Many clinical trials have been conducted to investigate the efficacy of mTOR inhibitors in the treatment of cancer, especially everolimus and LY3023414. Everolimus is an oral rapamycin analog, and its monotherapy showed an encouraging clinical benefit in patients with recurrent EC [192]. Although in recurrent ovarian, peritoneal, and fallopian tube cancers, everolimus did not distinctly improve the response when combined with bevacizumab [193], it is well tolerated at fully approved doses and is effective in patients with ER- and/or PR-positive ovarian or endometrial cancers when combined with aromatase inhibitor (anatrozole) [194]. Thus, everolimus-based combination therapies exhibited a promising effect and need to be further explored in future clinical trials. LY3023414 is a dual PI3K/mTOR inhibitor and exhibits a manageable safety profile and modest single-agent effect for advanced EC patients [195].

Gedatolisib (PF-05212384) demonstrated moderate activity with acceptable tolerability in patients with recurrent EC, as evaluated in a phase II study (NCT01420081) [196]. Ridaforolimus showed encouraging activity in advanced EC but was associated with significant toxicity [197], while in recurrent or metastatic EC, it had modest activity and was reasonably tolerated [198].

Temsirolimus exhibits encouraging antitumor effect in EC, and the responsiveness of chemotherapy-naive patients is more favorable than chemotherapy-treated patients [199], however, in both CC and persistent/recurrent EOC it shows modest activity as a single agent [200, 201]. The PI3K inhibitor buparlisib combined with the MEK1/2 inhibitor trametinib shows promising antitumor activity for KRAS-mutant OC [202].

### Notch signaling and targeted inhibitors

Notch signaling is evolutionarily conserved and essential for adult tissue homeostasis and the progression of multiple cancer types. The pathway is comprised of five canonical Notch ligands (DLL1, DLL3, DLL4, Jagged1, and Jagged2) and four Notch receptor paralogs (Notch1/2/3/4) [203]. Upon ligand binding, the Notch receptor is cleaved in the extracellular domain and then trans-endocytosed by the ligand-expressing cell. Following the second cleavage caused by γ-secretase activity, the intracellular fragment of Notch (ICN) is released and allowed to nuclear translocation where it associates with the CBF1/Su(H)/Lag-1 (CSL) transcription factor complex, resulting in subsequent activation of the target genes (*MYC*, *p21*, and *HES* family members) [203, 204].

As one of the most active pathways in cancer cells, Notch signaling plays a crucial role in the linkage between angiogenesis and CSC self-renewal [205]. In CD133^**+**^ EC cells, blocking the Notch pathway enhanced the suppressive effects of EGFR inhibitors on cancer cell proliferation [206]. Thus, targeting Notch signal transduction is a promising pharmacological intervention to eradicate endometrial CSCs. While numerous Notch inhibitors have been developed and investigated for cancer therapies, only RO4929097 has been explored in clinical research. It is a gamma-secretase inhibitor with minimal single-agent activity for patients with platinum-resistant OC [207].

### JAK/STAT signaling pathway and targeted inhibitors

Abnormally constitutive activation of the JAK/STAT pathway is associated with tumor progression and a poor prognosis in OC [208]. JAK/STAT pathway mediates tumor progression through the induction of numerous proteins and cytokines attributed to cell proliferation, stemness, and evasion from antitumor immunity [209]. By inducing the synthesis of immunological checkpoints (e.g., PD-1, PD-L1, PD-L2, and CTLA-4), STAT is a major contributor to the resistance of radiation therapy and chemotherapy and the failure of targeted immunotherapies [210].

Ruxolitinib, a JAK1/2 inhibitor, was demonstrated to be active in various peripheral T cell lymphoma subtypes [211]. Though the JAK1 inhibitor itacitinib showed manageable safety and clinical activity when combined with chemotherapy in patients with advanced solid tumors, it was terminated for negative phase III results in patients with previously treated advanced pancreatic cancer [212]. JAK2 inhibitor TG101348 is well tolerated and provides durable therapeutic benefit for myelofibrosis patients [213]. However, these inhibitors have not been evaluated in gynecological cancers.

### Wnt/β-catenin pathway

A large number of genes related to tumor formation and progression have been identified to be transcriptionally activated by Wnt/β-Catenin signaling [214]. In CD133/CD44^**+**^ endometrial CSCs, the activation of Wnt signaling was complicated by the upregulation of stemness-associated genes (such as *SOX2*, *OCT4*, and *NANOG*), which can be decreased by targeting SMOC-2 [215].

Aberrant activation of Wnt/β-Catenin signaling, especially mutation of *CTNNB1*, *AXIN*, and *APC*, was observed in endometrial and mucinous EOC subtypes [216]. The Wnt pathway is essential for the occurrence, progression, metastasis, angiogenesis, and chemoresistance of OC due to its crucial roles in CSC self-renewal, EMT, invasion capabilities, and tumor immunity suppression [217].

## Poly (adenosine diphosphate-ribose) polymerase inhibitors

### The DNA damage response in gynecological cancers

The DNA damage response (DDR) induces programmed cell death in the presence of severe DNA damage in reproducing cells by stopping cell cycle progression, which makes it easier to repair DNA lesions to stop mutagenesis and maintain genomic stability [218].

Localized DDR activation is triggered by HPV replication centers in CC [219, 220], and there is evidence that HPV E7 oncoprotein and HPV genomic integration may induce the upregulation of DDR proteins [221, 222]. Compared to their HPV-negative counterparts, HPV-positive cancer cells are more radiosensitive; however, resistance to DNA-damaging therapy still exists as shown by the poor prognosis of HPV-positive advanced CC [223, 224]. Therapeutic regulation of the DDR is an appealing technique to improve treatment response. Further research is required to understand how HPV affects the DDR and how it affects the *in vivo* sensitivity of HPV-associated malignancies to DNA-damaging agents.

Many aspects of OC biology are affected by abnormal DDR and DNA repair deficiency, including tumor initiation, progression from low-grade towards high-grade phenotype, responsiveness to chemotherapy, and development of chemoresistance. Numerous single nucleotide polymorphisms in genes linked to the DNA repair and DDR are expected to enhance the risk of OC [225].

PUMA, a new agent with therapeutic potential for OC, produces DNA breaks and activates kinases involved in DNA damage. Therefore, it was proposed to enhance the DDR in OC cell lines, increasing the rate of apoptosis [226, 227]. Upregulation of SEI1 (also known as TRIPBr1 and SERTAD1) causes increased DNA strand breaks, and DDR proteins were significantly downregulated and the number of micronuclei was greatly decreased upon SEI1 knockdown. Accordingly, SEI1 controls genomic stability by altering the DDR when cancer cells progress towards malignant phenotype [228].

Platinum–DNA damage tolerance and cisplatin sensitivity were found to be highly correlated [229]. OC patients had higher levels of DNA damage and a failure in DNA repair than did healthy individuals. Additionally, platinum-sensitive individuals had higher DNA damage levels than platinum-resistant patients [230].

A lack of DNA MMR proteins, such as MLH1, MSH2, MSH6, and/or PMS2, is seen in 30–40% of endometrioid tumors. This may be caused by germline mutations like in Lynch syndrome, or *MLH1* promoter hypermethylation [231]. A possible role for therapies that target DNA repair processes and take advantage of an already underdeveloped repair pathway is suggested by the loss of MMR proteins, which are important in the repair of DNA single-strand breaks (SSBs). Additionally, MSI is triggered by mismatch repair-deficient (dMMR) and results in a phenotype that would be more vulnerable to checkpoint inhibition. There are significant indicators for prospective targeted therapy, such as PARP inhibition, in this molecular subtyping.

### PARP inhibitors in gynecological cancers

**Fig. 3:**
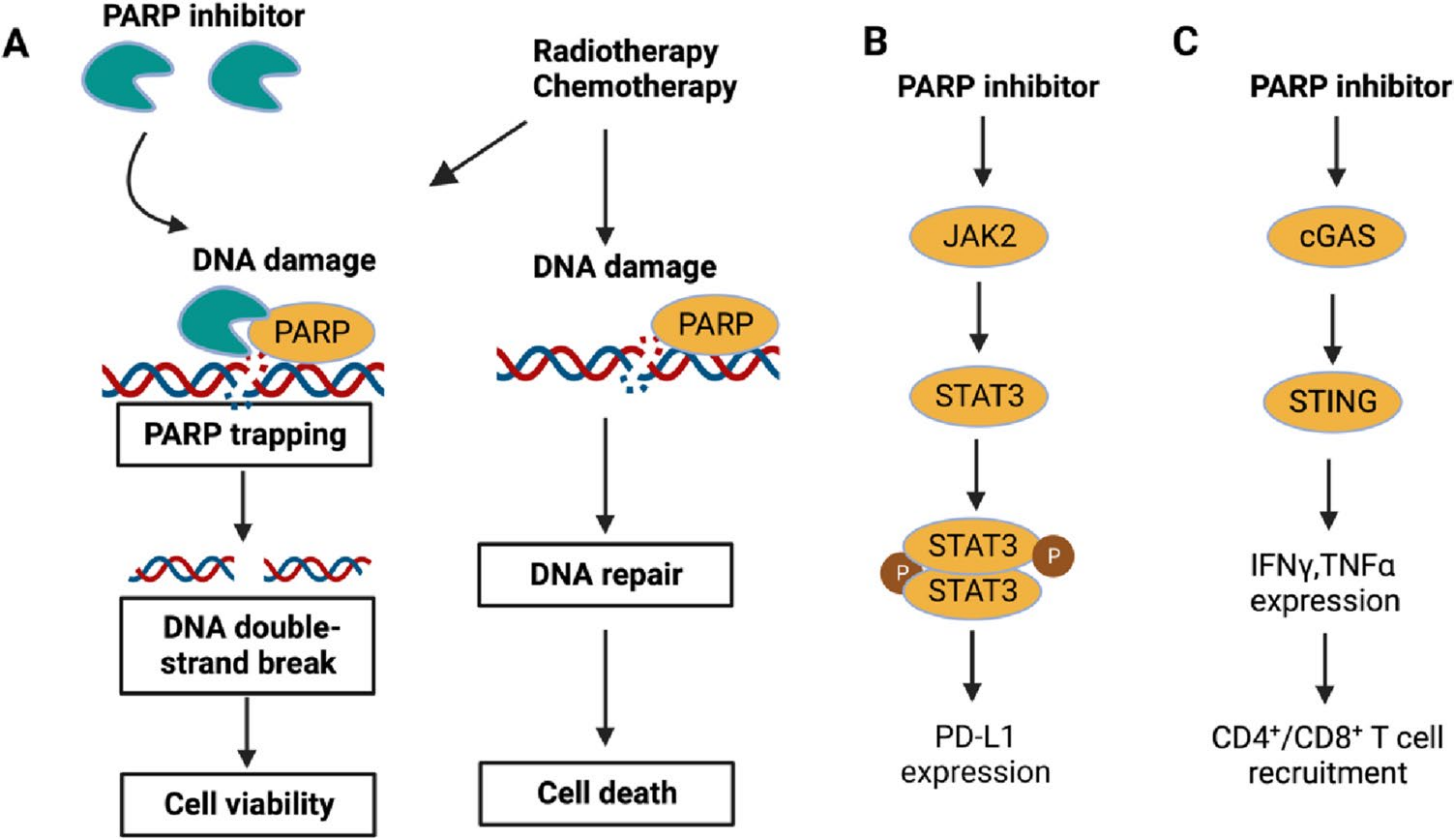
The antitumor mechanisms of PARP inhibitors. (**A**) The PARP inhibitor was devised to dampen the repair of DNA damage, which results in “synthetic lethality” in *BRCA* mutant cancer cells. (**B**) PARP inhibition can induce PD-L1 expression via JAK2/STAT3 pathway in solid tumors. (**C**) In *BRCA1*-deficient ovarian cancer, PARP inhibitor can increase the number of intratumoral CD4^+^ and CD8.^+^ T cells through a STING-dependent antitumor immunity (created with BioRender.com)

The detection and repair of SSBs depend on PARP enzymes, and PARPis inactivate these enzymes which allows the persistence of spontaneous SSBs and inhibits the release of PARP from DNA (“PARP trapping”). Both processes result in double-strand breaks (DSBs), collapsed replication forks, and persistent SSBs. DSBs can be repaired in two ways: through homologous recombination repair (HRR) or nonhomologous end-joining (NHEJ). NHEJ is an error-prone process that results in genetic instability, whereas homologous recombination repairs DNA with high fidelity [232]. As a result of the functional termination of two DNA repair processes in cells with HRR deficits (HRD), PARPis cause “synthetic lethality,” which results in a dependence on NHEJ and ultimately cell death brought on by the accumulation of genetic damage [233, 234]. Therefore, malignancies with HRD respond particularly well to PARPis. HRR depends on BRCA proteins, and germline *BRCA* (g*BRCA*) mutations and somatic *BRCA* (s*BRCA)* mutations can cause malignant transformation and make cancers vulnerable to PARPis [235]. The HRR pathway also experiences additional genetic and epigenetic alterations, providing other potential targets for PARP inhibition. In BRCA1-deficient OC, inhibition of PARP could induce PD-L1 expression via JAK2/STAT3 pathway in solid tumors and increase the number of intratumoral CD4^+^ and CD8^+^ T cells through a STING-dependent antitumor immunity [236, 237] (Fig. 3). PARPis and the ongoing clinical trials are collected in Table 1.

**Table 1:** PARPis and ongoing clinical trials in gynecological cancers

PARPi	Combination with	Clinical trial number	Conditions or diseases	Phase	ECD
Niraparib	Copanlisib	NCT03586661	Recurrent endometrial and ovarian cancers	I	December 31, 2022
Rucaparib	Nivolumab	NCT03572478	Advanced/recurrent endometrial cancer	II	March 9, 2021
Olaparib	Durvalumab/Capivasertib	NCT03660826	Recurrent, persistent, or metastatic endometrial cancer	II	April 15, 2024
Rucaparib	Bevacizumab	NCT03476798	Recurrent carcinoma of the cervix or endometrium	II	February 2023
niraparib	Pelvic radiotherapy	NCT03644342	Metastatic cervical cancer	I/II	March 2, 2026
Olaparib	MEDI4736	NCT02734004	Advanced solid tumors	I/II	December 30, 2022
Olaparib	Cediranib	NCT03278717	Platinum-sensitive ovarian, fallopian tube, or peritoneal cancer	III	December 2023
Olaparib	Cediranib	NCT02502266	Recurrent platinum-resistant or platinum-refractory ovarian, fallopian tube, or primary peritoneal cancer	II/III	June 30, 2023
Olaparib	AZD6738 (ATR inhibitor drug)	NCT03462342	Recurrent ovarian cancer	II	December 31, 2022
Olaparib	AZD6738 (ATR inhibitor drug)	NCT04065269	Gynecological cancers with ARId1A Loss	II	March 2023
Olaparib	Selumetinib	NCT03162627	Endometrial, ovarian, and other solid tumors with Ras pathway alterations, and ovarian tumors with PARP resistance	I	August 30, 2026
Niraparib	Copanlisib	NCT03586661	Recurrent endometrial, ovarian, primary peritoneal, or fallopian tube cancers	I	December 31, 2022
Olaparib	Adavosertib/Ceralasertib	NCT03579316	Recurrent ovarian, primary peritoneal, or fallopian tube cancers	II	December 30, 2023

*ECD*, estimated completion date

#### PARPis in endometrial cancer

Loss function of PTEN, the most frequent mutation in EC, can result in inadequate repair of DSBs and consequent sensitivity to PARPis [238, 239]. Preclinical research has indicated a potential benefit of PARPis in PTEN-deficient cell lines and tumor models [238, 240], and PI3K blockade can sensitize cells to PARPis [239, 241].

The genetic similarities between high-grade serous ovarian cancer and serous endometrial cancer imply that serous endometrial tumors with high copy number, even those lacking *BRCA* mutation, may also benefit from PARPis. One phase I trial (NCT03586661) is assessing niraparib, a PARPi, with the PI3K inhibitor copanlisib in patients with recurrent endometrial and ovarian malignancies, and other clinical trials are actively evaluating the antitumor effect of a single-agent PARPi.

It is acknowledged that tumors with a lot of mutations also have many neoantigens and PD-1 expression, therefore, immune checkpoint therapy may strengthen the efficacy of PARPis in endometrial malignancies with polymerase-epsilon (POLE) mutation or high microsatellite instability (MSI-H) [231]. Accordingly, several associated clinical trials have been established. A phase II DOMEC trial (NCT03951415) conducted the synergetic efficacy of PARPi olaparib and durvalumab (a selective monoclonal antibody that blocks PD-L1 with high-affinity) in metastatic or recurrent EC patients, and the results showed that it was well tolerated, but did not meet the prespecified 50% 6-month progression-free survival [242].

Similar to OC, EC may benefit from the combination of PARPi and antiangiogenic therapy. In cases of recurrent and/or refractory EC, combinations of rucaparib and bevacizumab (NCT03476798), and olaparib and cediranib (NCT03660826) are being studied.

#### PARPis in cervical cancer

Preclinical research has demonstrated that PARPis boost apoptotic response and make CC cells more sensitive to cisplatin [243–245]. Preclinical research has been done on PARPis in CC, several clinical trials are currently being conducted [246].

The utility of PARPis in advanced-stage CC was examined in two GOG/NRG oncology studies [247, 248]. For the treatment of patients with advanced, persistent, or recurrent CC, PARPi veliparib was coupled with paclitaxel and cisplatin in a phase I trial. The median PFS was 6.2 months (95% CI 2.9–10.1 months), and OS was 14.5 months (95% CI 8.2–19.4 months). The only grade 3 and 4 adverse effects were dyspnea and neutropenia, the maximum tolerable dose was not reached, and the ORR for patients with measurable diseases was 34%. The researchers concluded that the combination therapy was safe and practical in persistent and recurrent CC [247]. In the second clinical research examining the efficacy and tolerance of PARPi veliparib and topotecan (a potent anticancer camptothecin analog), twenty-seven patients with persistent or recurrent CC were examined. Grades 3 and 4 toxicities frequently occurred, including anemia (59%), thrombocytopenia (44%), leukopenia (22%), and neutropenia (19%). Only 2 patients (7%) showed signs of partial response, while 4 experienced disease progression after 6 months of treatment. Overall, this research showed that veliparib with topotecan had serious side effects with little clinical activity. However, a subset of patients with low PARP-1 levels on immunohistochemical staining showed noticeably greater PFS and OS, indicating that PARP-1 may be a viable biomarker for identifying patients who may benefit from this treatment [248].

Additional clinical trials are currently being conducted to further investigate the efficacy of PARPis combination therapies in CC. In patients with metastatic, recurrent, or persistent gynecological cancers, several combination therapies of PARPi are being researched [249]. In phase II research called Clovis-001 (NCT03476798), the combination of rucaparib and bevacizumab is being tested in recurrent cervical or endometrial cancer. PARPis make cancer cells more radiosensitive because radiation causes DNA damage, which may theoretically be increased by preventing DNA repair pathways. Reduced survival was observed in pancreatic cancer cell lines treated with radiation in combination to rucaparib [250]. In the phase I/II research NIVIX (NCT03644342), the combination of niraparib with pelvic radiation is being studied in metastatic stage IV invasive CC.

#### PARPis in ovarian cancer

Multiple clinical trials have proved that PARPis function as a feasible therapy option in patients with and without HRD, manifested by significantly prolonged PFS [251]. Three PARPis (olaparib, rucaparib, and niraparib) have been approved for OC treatment by the FDA and EMA [252]. Studies on other PARPis, such as veliparib and talazoparib, have exhibited encouraging clinical outcomes and will soon gain licensure (NCT01472783, NCT02470585, NCT01540565, NCT01286987).

In a phase II (NCT00628251) randomized research assessing olaparib plus PLD in patients with g*BRCA* mutated platinum-resistant or partially platinum-sensitive relapsed OC, researchers compared the efficacy of PARPis as monotherapy and that of chemotherapy, and they found that there was no distinctive difference in ORR or PFS [253]. Later the phase III trial SOLO3 (NCT02282020) was conducted to assess olaparib monotherapy versus nonplatinum chemotherapy (PLD, paclitaxel, gemcitabine, or topotecan) in patients with g*BRCA* mutated platinum-sensitive relapsed ovarian cancer (PSROC) who had undergone two or more cycles of platinum-based chemotherapy. The ORR was much higher in the olaparib group than that of chemotherapy group in the total 266 patients (72.2% vs. 51.4%; *P* = 0.002). As for the patients who had received 2 prior lines of platinum treatment, the ORR was 84.6% with olaparib and 61.5% with chemotherapy. Olaparib considerably outperformed chemotherapy in terms of PFS (13.4 v 9.2 months; *P* = 0.013). Adverse events were consistent with the established safety profiles of olaparib and chemotherapy [254]. It should be highlighted that although olaparib resulted in statistically significant and clinically relevant improvements, the control arm in this trial was a nonplatinum therapy for platinum-sensitive patients; consequently, the practicality for clinical application is doubted.

Based on the condition of g*BRCA* mutation (g*BRCA*m) and the level of loss of heterozygosity (LOH), the phase II trial ARIEL2 (NCT01891344) assessed the efficacy of rucaparib monotherapy in patients with three different types of HRD (g*BRCA*m, non-g*BRCA*m/LOH high, and non-g*BRCA*m/LOH low) and the biggest clinical improvements were observed in the g*BRCA*m cohort with the highest PFS and ORR [255, 256].

Olaparib was tested in a phase II (NCT00679783) multicenter, nonrandomized trial in patients with advanced high-grade serous and/or poorly differentiated OC. Among the 63 patients who had target lesions, the ORR for g*BRCA*m cohort was 33% and for non-g*BRCA*m cohort it was 4% [257]. Based on the QUADRA trial (NCT02354586), niraparib was approved in 2020 as the maintenance treatment for recurrent OC patients regardless of genetic profiles [258].

The expressions of PD-1 and neoantigen for possible immune system recognition in g*BRCA*m or HRD tumors make it possible to improve anticancer efficacy through combination therapy of PARPis and immune checkpoint inhibitors (ICIs). Currently, many ongoing clinical research are being conducted to assess the impact of ICIs with PARPis on OC patients [259]. A Phase I/II Study is actively evaluating the efficacy of MEDI4736, an anti-PD-L1 antibody, in combination with olaparib for advanced OC patients (NCT02734004).

PARPi combined with antiangiogenic treatment is another area with potential value. It has been validated that hypoxia caused the defects in HRR genes, including *BRCA* [260]. When compared to bevacizumab monotherapy, olaparib combined with bevacizumab maintenance produced a better PFS in newly diagnosed, advanced OC patients, according to a randomized, double-blind PAOLA-1/ENGOT-ov25 trial (NCT02477644) [261]. Niraparib plus bevacizumab significantly improved PFS compared to single-agent niraparib (11.9 vs. 6.4 months; *P* = 0.0001) (NCT02354131), while olaparib and cediranib together improved PFS by 8.7 months (NCT01116648) in patients with PSROC. These findings suggest that this synergetic strategy is effective in patients with PSROC independent of *BRCA* status and that the anti-angiogenesis drugs may be especially significant for g*BRCA* mutated tumors. Accordingly, the phase III trials (NCT03278717, NCT02502266) are now being carried actively to further evaluate the effects of these combination therapies on recurrent OC.

Combination strategies that focus on other genes involved in the HRR pathway are also gaining popularity. It has been validated that PI3K inhibition could decrease the expression of BRCA and improve the efficacy of PARPis [262]. The recommended dosage of the mTOR inhibitor vistusertib plus olaparib showed minimal clinical benefit in OC patients, with 20% RR and 15% RR observed in each group of a phase I trial (NCT02208375). In a phase I research (NCT01623349), olaparib was also examined in combination with the pan-PI3K inhibitor buparlisib in patients with advanced OC. The RR was 29%, and all the patients who responded harbored high-grade serous histology and the majority (8 out of 12 responders) had g*BRCA*m. In addition, there was no difference in response to the combination regimen between patients who had platinum-sensitive or platinum-resistant disease [263]. Olaparib and alpelisib, a PI3K-alpha inhibitor, were combined in a multicenter, open-label, phase Ib trial in the 28 recurrent EOC patients with high-grade serous histology, and 36% of participants achieved partial response and 50% showed stable disease [264]. Based on the above research, regimen combining olaparib and PI3K inhibitors is feasible and exhibits promising preliminary clinical evidence particularly in patients with advanced EOC, which warrants further investigation.

Furthermore, multiple phase I studies are now being conducted in patients with OC to evaluate the combination therapies of PARPis with TKIs that target ATR, MEK1/2, PI3K, WEE1, and/or PI3K (NCT03162627, NCT04065269, NCT03579316).

## Immunotherapeutic strategies for gynecological cancers

Infiltrated immune cells serve a crucial role in the progression of tumors and co-constitute heterogeneous microenvironment by combining different cells and extracellular components. It is acknowledged that the numbers of neutrophils, macrophages, and dendritic cells (DCs) are significantly increased in EC, while NK cells are significantly decreased [265]. The fact that tumor-associated macrophages (TAMs) but not regulatory T cells (Tregs) have been proved to be correlated with a poor prognosis and lymph node metastasis of EC [266] together with the preferential enrichment of macrophages and exhausted CD8^+^ T cells suggests an immunosuppressive microenvironment in EC [267].

Infiltrating macrophages act as a driver of type I EC by sensitizing EC cells to estrogen through upregulating estrogen receptor alpha expression, and it was discovered to be a prognostic biomarker of cancer progression [268]. CCL2-facilitated macrophage infiltration has also been confirmed by *in vitro* studies [269]. Macrophages also promote metastasis through EMT via CCL18-activated PI3K/AKT/mTOR signaling in EC [270]. In addition, infiltrated macrophages supply interleukin (IL)-8, which might be associated with myometrial invasion and angiogenesis in EC [271].

Cytotoxic T lymphocytes are essential for tumor control, but their functions are compromised by cancer cells and Tregs. The suppression of T cell-mediated antitumor immunity was suggested to be associated with progression and development of EC through B7-H3 [272]. Higher levels of immunosuppressive cytokines, including TGF-β, were involved in EC and CD8^+^ T cell-mediated cytotoxic capacity was suppressed by downregulating intracellular cytolytic molecules [273]. Tregs were also reported to induce tolerance of immune therapy in EC [274, 275], and the number of FoxP3^N^ Tregs was increased in dMMR EC [276]. The number of CD8^+^/CD4^+^ T cells and Tregs, and Treg/CD8^+^ and Treg/CD4^+^ ratios were significantly higher in patients with advanced poorly differentiated EC. Accordingly, the disease-free survival of patients with higher Tregs and Treg/CD8^+^ ratios was significantly worse than that of patients with lower Tregs and Treg/CD8^+^ ratios [274].

Given the molecular mechanisms and findings from multiple clinical research aiming at evaluating the antitumor efficacy of several promising ICIs (Table 2), the immunotherapeutics exert exciting new frontiers for patients with gynecological cancers.

**Table 2:** Immune checkpoint inhibitors and clinical trials in gynecological cancers

Intervention	ID	Cancer/condition	Targets	Phase	ORR (%)	mPFS (mon.)	mOS (mon.)	SAEs (%)	Refs
Pembrolizumab	NCT02628067 KEYNOTE-028	EC/advanced	PD-1	Ib	13.0%	1.8	−	54.2%	[282]
Avelumab	NCT02912572	EC/dMMR	PD-	II	26.7%	4.4	−	71%	[363]
		EC/pMMR	L1		6.25%	1.9	6.6		
Durvalumab	NCT03015129	EC/pMMR	PD-L1	II	3%	1.8	12. 1	93%	[364]
		EC/dMMR			47%	8.3	−		
Durvalumab + Olaparib	NCT03951415	EC/metastatic or recurrent	PD-L1 + PARP	II	16%	3.4	8.0	88%	[242]
Dostarlimab	NCT02715284	EC/dMMR	PD-1	I	42.3	−	−	−	[365]
Dostarlimab	NCT02715284	EC/dMMR MSI-H	PD-1	I	43.5%	−	−	−	[290]
		EC/MMRp MSS			14.1%				
Lenvatinib + Pembrolizumab	NCT02501096	EC/advanced	Multikinase + PD-1	Ib/II	38.0%	7.4	16.7	66.9% (grades 3–4)	[366]
Lenvatinib + Pembrolizumab	NCT03517449	EC/pMMR	Multikinase + PD-1	III	31.9%	6.6	17.4	88.9% (grades ≥ 3)	[292]
Pembrolizumab	NCT02628067	EC/dMMR MSI-H	PD-1	II	48%	13.1	−	12% (grades 3–4,)	[367]
Pembrolizumab	NCT02054806 KEYNOTE-028	OC/advanced	PD-1	Ib	11.5%	1.9	13.8	73.1%	[368]
Pembrolizumab	NCT02674061	OC/epithelial recurrent	PD-1	II	19.0%	−	−	61.9%	[369]
	KEYNOTE-100								
Nivolumab	NCT02498600	OC/recurrent or persistent	PD-1	II	12.2%	2	−	33%(grades ≥ 3)	[370]
Nivolumab + ipilimumab			PD-1 + CTLA-4		31.4%	3.9	−	49%	
Nivolumab + bevacizumab	NCT02873962	OC/relapsed	PD-1 + VEGF	II	21	44	−	−	[371]
Atezolizumab + bevacizumab	NCT01633970	OC	PD-L1 + VEGF	Ib	15%	4.9	10.2	−	[372]
Cemiplimab	NCT03257267	CC/recurrent	PD-1	phase 3	16.4%	2.8	12.0	2.8	[373]
Balstilimab	NCT03104699	CC/Recurrent and/or metastatic	PD-1	II	−	−	−	71.4	[374]
Nivolumab	NCT02257528	CC/persistent or recurrent	PD-1	II	4	** − **	** − **	84%	[375]
	NRG-GY002							24%(grade ≥ 3)	
Balstilimab + zalifrelimab	NCT03495882	CC/recurrent and/or metastatic	PD-1 + CTLA-4	II	25.6%	2.7	12.8	20.0% (grade ≥ 3)	[297]

*dMMR*, mismatch repair-deficient, *pMMR*, mismatch repair-proficient, *MSS*, microsatellite stable, *MSI*, microsatellite instable, *POLE*, polymerase-ε, *SAEs*, serious adverse events

### Immune checkpoint inhibitors for gynecological cancers

#### PD-1/PD-L1 blockade

PD-1 is widely expressed in activated T cells, NK cells, B lymphocytes, macrophages, DCs, and monocytes [277, 278]. The activity of PD-1 and its ligands (PD-L1 and PD-L2) is responsible for regulating T cell activity, activating apoptosis of antigen-specific T cells and inhibiting Tregs apoptosis [277]. The PD-1/PD-L1 axis, a target of immunotherapy, plays a pivotal role in promoting immune self-tolerance and cancer escape. It was found that 61.3% of EC were PD‑1 positive, which was almost exclusively in tumor‑infiltrating immune cells [279]. Correspondingly, PD‑L1 was positive in 14.3% of normal endometria and in 17.3% of EC tissues, while PD‑L2 was positive in 20.0% of normal endometria and 37.3% of EC tissues [279].

As a monoclonal IgG4 kappa-isotype antibody against the PD-1 receptor, pembrolizumab was approved for dMMR or MSI-H EC patients and recurrent or metastatic CC patients [280, 281]. In the KEYNOTE-028 study, pembrolizumab monotherapy demonstrated durable antitumor activity in a cohort of patients with PD-L1-positive, advanced EC. The ORR was 13.0% and the median PFS was 1.8 months, with adverse events including fatigue, pruritus, pyrexia, and decreased appetite [282]. In MSI-H or dMMR advanced EC, PD-1 inhibitors avelumab and durvalumab have shown a response rate of 27% and 43%, respectively. While, dostarlimab and pembrolizumab have shown a response rate of 49% and 57%, respectively [283]. Pembrolizumab treatment in patients with recurrent CC also shows considerable clinical benefits with manageable adverse effects [284, 285]. Dostarlimab has recently been approved in the EU and USA for the treatment of patients with dMMR recurrent or advanced EC [286]. The protective effect of dostarlimab is still being evaluated in ongoing clinical trials in advanced CC patients and recurrent OC patients who are not suitable for platinum treatment (NCT03833479 and NCT04679064, respectively) [287, 288]. As for MSI EC, dostarlimab showed a higher affinity against PD-1 than pembrolizumab with encouraging clinical activity, manifested by the preliminary results [289], and it exhibited significant antitumor activity in both dMMR/MSI-H and mismatch repair-proficient (pMMR)/microsatellite stable (MSS) EC with a manageable safety profile, based on the results from a phase I, single-arm study [290].

**Fig. 4:**
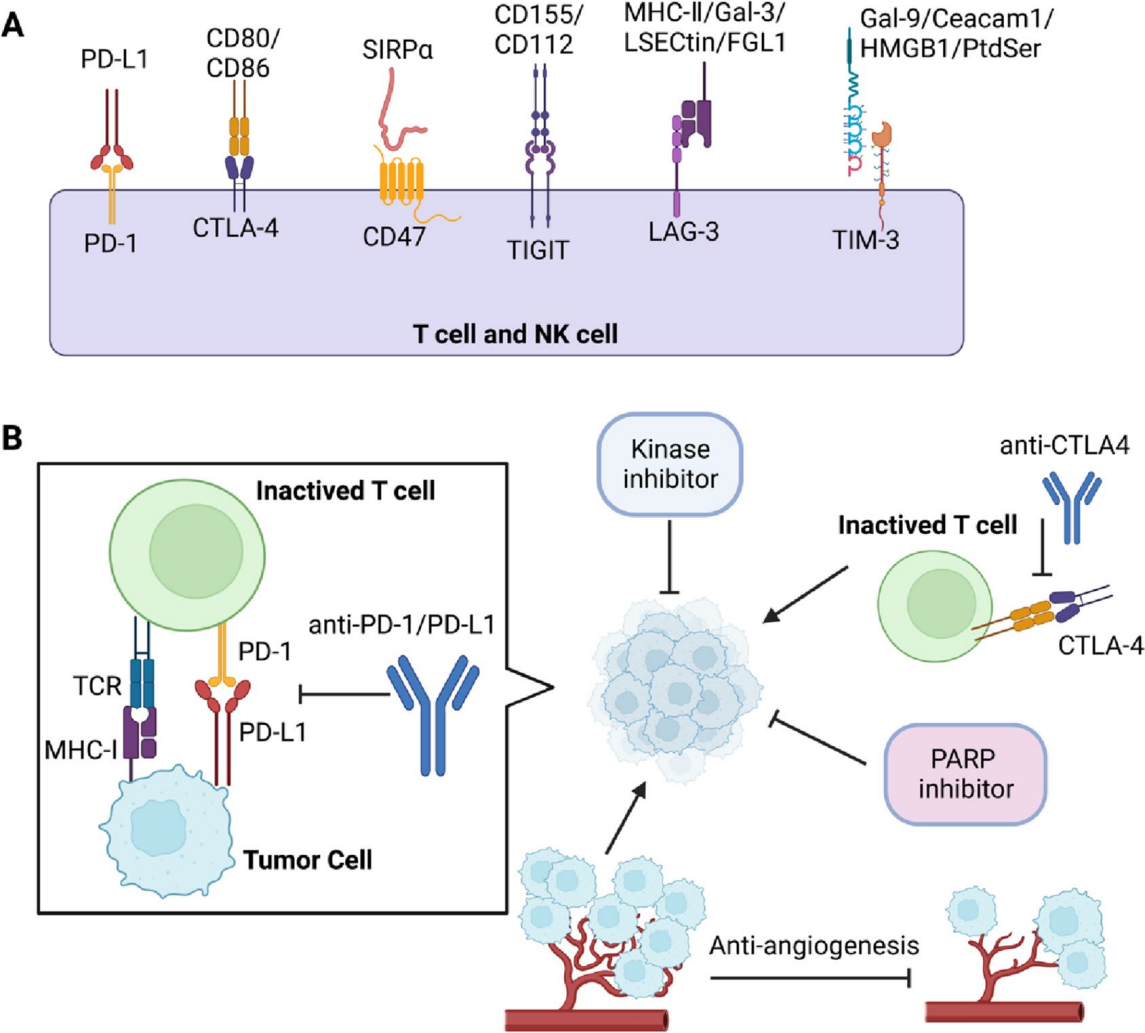
Targeted immunotherapies and the anti-PD-1/PD-L1 combination strategies in gynecological cancers. (**A**) The promising immunotherapeutic targets for gynecological cancers. (**B**) Blockade of the PD-1/PD-L1 checkpoint promotes anti-tumor T cell immunity. The synergistic antitumor strategies of anti-PD-1/PD-L1 in combination with PARP inhibitors, anti-CTLA4 antibody, kinase inhibitors, or angiogenesis inhibitors involved in clinical trials (created with BioRender.com)

PD-1/PD-L1 blockade has also been investigated for combination therapies in gynecological cancers (Fig. 4). Preclinical studies demonstrated that lenvatinib, a multiple receptor TKI targeting VEGF and FGF receptors, increased IFN-γ^+^ and granzyme B^+^ CD8^+^ T cells, which improved cancer immunotherapy when combined with PD-1 blockade, and its combination therapy with pembrolizumab has approved by FDA for patients with advanced EC [291]. In the 309/KEYNOTE-775 study, patients treated with pembrolizumab plus lenvatinib showed longer PFS (6.6 months) than those in the chemotherapy arm (3.8 months). However, in addition to adverse events, a higher occurrence rate (88.9%) was found in the combination treatment group, suggesting palliative treatment [292]. The combination of PD-L1 blockers with PARPis has been demonstrated to exert a synergistic effect in patients with recurrent EC [293].

#### CTLA-4 blockade

CTLA-4 is expressed exclusively in T cells, where it dampens the activation of T cells by outcompeting CD28 in binding to CD80 and CD86, as well as actively delivering inhibitory signals to the T cells [294]. The expression of CTLA-4 seems to be higher than that of PD-L1 and is associated with a low CD4^+^/CD8^+^ ratio and high tumor grade in EC [295].

CTLA-4 blocking antibody (MDX-CTLA4) stimulated extensive tumor necrosis with lymphocyte and granulocyte infiltrates in vaccinated patients with metastatic OC [296]. In a phase II trial (NCT03495882), the CTLA-4 antibody zalifrelimab was combined with a PD-1 antibody (balstilimab) in patients with recurrent and/or metastatic CC, and the ORR was 25.6% [297]. In recurrent BRCA-deficient OC patients, a combination of PARPi and CTLA-4 blockade (tremelimumab) led to decreased tumor size with grade 1 and 2 toxicities [298].

#### CD47 blockade

**Fig. 5:**
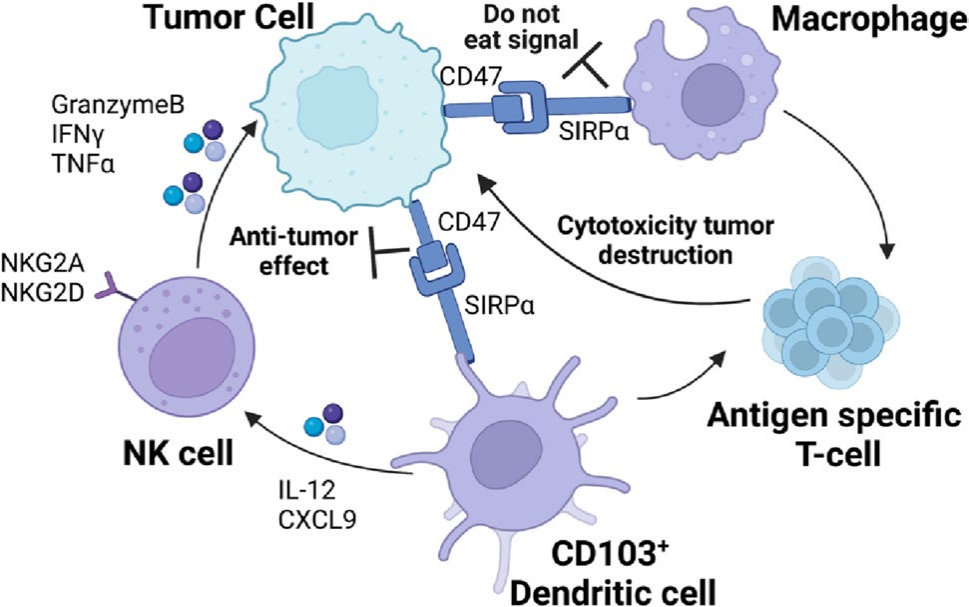
Mechanisms of CD47 mediated immune tolerance in tumor microenvironment. CD47 expressed in cancer cells activates SIRPα on macrophages, which delivers a “do not eat me signal.” Furthermore, the interactions between CD47 on tumor cells and SIRPα expressed in CD103.^+^ dendritic cells contribute to restrict NK cell recruitment which elicit anti-tumor effect by orchestrating the expression of granzyme B, IFN-γ, TNF-α, NKG2A, and NKG2D (created with BioRender.com)

CD47 protein, expressed in both healthy and cancer cells, plays a crucial role in blocking the cytotoxic activity of myeloid cells by delivering a “do not eat me signal” upon binding to the signal-regulatory protein alpha (SIRPα) receptor on macrophage cells (Fig. 5) [299].

CD47 cannot be observed in the normal endometrium, but its expression is increased along with the progression of endometrial hyperplasia to carcinoma, and elevated CD47 expression was also associated with poorer prognosis and higher clinicopathological grade of EC [300]. In EC, CD47 acts as an antiphagocytic signal that promotes tumor resistance against tumor-associated macrophages, and CD47 blockade increased the infiltration of macrophages *in vivo* and promoted phagocytosis of EC cells by M2 macrophages [301]. Recent studies in endometriosis revealed that targeting CD47 increased macrophage phagocytosis and induced apoptosis of ectopic endometrial stromal cells [302]. These results suggest a dual mechanism of CD47-SIRPα signaling in eradicating targeted cells.

Preclinical research has demonstrated that targeting CD47 is a promising approach for improving OC treatment both as a single-agent therapy or in combination with another agent [303]. CD47/SIRPα inhibitors include magrolimab (Hu5F9-G4), TTI-621, ALX148 and several small-molecule inhibitors, such as RRx-001 [304–306]. Although magrolimab has been approved for the treatment of hematopoietic malignancies and several other CD47/SIRPα inhibitors have been assessed in many advanced cancers [307–310], clinical data about the efficacy of CD47/SIRPα inhibitors in patients with gynecological cancers are rare. Herein, in a first-in-class phase I trial of Hu5F9-G4 in patients with advanced cancers, two patients with ovarian/fallopian tube cancers had partial remissions for 5.2 and 9.2 months and reductions of 50% and 44% in target lesions, respectively [311].

### Engineered immune-activating strategies for gynecological cancers

#### Oncolytic viral immunotherapy

Oncolytic therapy, specifically targeting cancer cells, has provided a revolutionary tool for converting “immune-cold” tumors to “immune-hot” tumors with antitumor responses. The cytotoxicity of oncolytic viruses occurs through a dual mechanism that is dependent on directly triggering tumor cell lysis and the induction of systemic antitumor immunity. For instance, oncolytic virus-induced tumor cell lysis may result in the release of a variety of tumor-associated antigens/neoantigens, danger-associated molecular patterns, and viral pathogen-associated molecular patterns to trigger inflammatory immune responses [312].

Intraperitoneal Olvi-Vec virotherapy, based on a modified vaccinia virus, showed promising safety and clinical activity in platinum-resistant or refractory OC patients through enhancing tumor infiltration of CD8^+^ T cells and activating tumor-specific T cells in peripheral blood [313]. The safety and efficacy of enadenotucirev, a tumor-selective adenoviral vector, were evaluated in platinum-resistant OC. Enadenotucirev plus paclitaxel demonstrated manageable tolerability and prolonged median PFS by increasing tumor immune-cell infiltration in platinum-resistant OC [314]. Oncolytic reoviral therapy was also evaluated in patients with recurrent ovarian, tubal, or peritoneal cancer. However, the addition of reovirus to paclitaxel did not sufficiently reduce the hazard of progression or death [315]. Oncolytic measles virus has been engineered to express carcinoembryonic antigen and showed dose-dependent biological activity in recurrent OC patients [316]. Adenovirus has also been reported to be feasible and safe for recurrent OC patients, suggesting a potential therapeutic option [317].

#### Adoptive cell therapies

Adoptive T cell therapy (ACT) has emerged as an effective and potentially curative therapy for malignancies, which utilizes either tumor-infiltrating lymphocyte (TIL)-derived T cells or T cells genetically modified to express tumor-recognizing receptors [318]. Engineered T cells for cancer therapy include antigen-specific transgenic T cell receptor T cells (TCR-T cells) and chimeric antigen receptor T cells (CAR-T cells).

In recent years, numerous clinical trials associated with TCR-T and CAR-T cells have been ongoing. Adoptive transfer of folate receptor-α-redirected autologous T cells showed preliminary activity in patients with recurrent OC [319]. Due to the lack of ideal tumor surface antigens, CAR-T cell therapy showed limited outcomes in treating solid tumors. In contrast, TCR-T cells identify intracellular and cell-surface antigens presented by the major histocompatibility complex (MHC) and thus have the potential to access many more target antigens than CAR-T cells, providing great promise in treating solid tumors [320].

Currently, numerous antigens have been applied for TCR-T-engineered cells in clinical trials, including HPV16-E6/E7, NY-ESO-1, MAGE-A3, MAGE-A4, and mesothelin. Antigens used as CAR-T therapeutic targets include mesothelin, CD70, CD22, CD133, GD2, PSMA, MUC1, MUC16, HER-2, nectin-4, FR-a, ALPP, B7-H3 and TnMUC1 [321]. Although ACT is a promising option for treatment of malignant tumors, several challenges warrant further investigation, such as severe adverse effects, loss of target antigen, and the short persistence of transferred T cells [321]. Engineered modifications and clinical trials need to be further explored to facilitate the use of ACT for gynecologic tumors.

Adoptive NK cell therapy has shown an ORR of 26.7% in gynecological cancers, suggesting a promising treatment modality [322]. The CAR strategy was also applied to NK cells and macrophages, which led to CAR-NK and CAR-M cell therapies. These approaches seem to be alternatives to T cells and safer than CAR-T cells [323, 324], while the promise and challenges of these therapies need to be extensively explored in gynecological cancers.

### Other emerging candidates for immunotherapy: TIM-3, LAG-3, and TIGIT

With the growing knowledge of effective biomarkers, coinhibitory receptors, such as TIM-3, LAG-3, and TIGIT, have been identified for their crucial roles in modulating T cell responses and maintaining immune homeostasis.

TIM-3 is a type I transmembrane protein expressed in T cells, NK cells, monocytes, macrophages, and DCs. Upon interaction with its ligand(s), TIM-3 acts as a negative regulator of antitumor immunity via T cell exhaustion and suppression of innate immune responses [325]. Plasma-soluble TIM-3 has emerged as an inhibitor of sepsis progression, which contrasts with membrane TIM-3 on monocytes [326]. Previous study has demonstrated that TIM-3 is commonly expressed in both MMR-intact and dMMR EC [327]. Therefore, inhibitors targeting TIM-3 may be a potential strategy for EC therapy.

A few clinical trials of TIM-3 inhibitors, such as LY3321367, sabatolimab, and LY3415244, have been performed in patients with advanced solid tumors. The combination therapies of LY3321367 or sabatolimab with anti-PD-L1 antibody both exhibited safety profiles and favorable pharmacokinetics, while sabatolimab, compared to LY3321367, showed a better antitumor activity when administered in combination with spartalizuma [328, 329]. LY3415244, a TIM-3/PD-L1 bispecific antibody, was reported to result in the development of clinically significant anaphylactic infusion-related reactions in 16.7% of patients and treatment-emergent antidrug antibodies in all patients, which suggests unexpected immunogenicity [330]. Thus, the bispecific TIM-3 and PD-L1 format exhibited better promise than monotherapy and combination therapy, while how to lower the immunogenicity risk requires further investigation.

LAG-3, an immunosuppressive checkpoint molecule, expressed in lymphocytes and its ligand GAL-3, has been identified on EC cells, particularly in nonmethylated dMMR cancers, supportting a role for immunotherapies targeting LAG-3 and/or GAL-3 in a subset of EC [331]. In an analysis of 26 cervical tumors, LAG-3 was detected in 65% of cases while PD-L1 in 85% of cases [332]. In OC, LAG-3 was negatively related to the infiltration of a specific CD8^+^ T cell subset [333].

Ieramilimab (LAG525) is an inhibitor of LAG-3 and according to phase I/II clinical trials, it was tolerated in advanced malignancies with no synergistic antitumor activity in combination with anti-PD-1 spartalizumab (PDR001) [334]. Treatment with the LAG-3 blocking antibody relatlimab plus the PD-1 blocking antibody nivolumab showed better median PFS (10.1 months vs. 4.6 months) and PFS (47.7% vs. 36.0%) than nivolumab monotherapy in patients with previously untreated metastatic or unresectable melanoma [335].

The T cell immunoglobulin and ITIM domain (TIGIT) is a well-known member of the immunoglobulin superfamily that is expressed exclusively on lymphocytes, particularly on CD4^+^ Tregs, CD8^+^ T cells, and NKs. Previous studies revealed that TIGIT blockage could lower the immunosuppression induced by CD4^+^ Tregs in OC mice models, boost functional responsiveness of NKs towards OC, and anti-TIGIT treatment also exerted promising antitumor effect, which suggests that inhibition of TIGIT is a potential therapeutic target in OC patients due to its effect on NK cell suppression and Treg activation [336, 337].

Recently, a phase I clinical trial of the anti-TIGIT antibody vibostolimab alone or in combination with pembrolizumab for patients with advanced solid tumors demonstrated that the combination therapy showed favorable antitumor effect [338].

Accordingly, TIM-3, LAG-3, and TIGIT are the potential targets of emerging immunotherapies especially for patients with advanced solid tumors but have not been well-studied in gynecological cancers, especially in the clinical setting. Therefore, the clinical impact of those targets via monotherapy or combination therapies warrants further investigation.

## Repurposing of existing drugs in gynecological cancers

Repurposing approved drugs to identify new pharmacological/therapeutic indications is increasingly becoming an attractive proposition because the usage of de-risked compounds requires lower overall costs and shorter development timelines [339, 340]. During the recent decades, several “old” drugs have been found to exert promising antitumor effect in EC.

Aspirin directly inhibits the enzyme cyclooxygenase (COX), and its primary effect is considered to be on the anucleate platelet by inhibiting COX-1 acetylation. While circulating platelets are validated to play a critical role in tumor metastasis via immune evasion. Up to now, a large body of evidence supports the preventive role of aspirin in the development and recurrence of cancers in the observational setting [341]. The STICs and STONEs trial (NCT03480776), a randomized phase II, double-blind, placebo-controlled trial, is currently ongoing to assess the effect of aspirin in the prevention of OC with *BRCA1* and *BRCA2* mutations.

Metformin is a first-line drug for type 2 diabetes and has beneficial effects on various metabolic syndromes. Most experimental data have revealed that it electively targets CSCs and acts together with chemotherapy to exert antitumor effects in different cancers mainly through AMPK activation and PI3K/AKT/mTOR inhibition. Given the available clinical findings and the molecular mechanisms, it also showed a potential role as an adjuvant therapy for EC [342, 343].

Quinacrine (QC) is an oral, inexpensive drug that was initially used extensively as an antimalarial drug, which exhibited significant antitumor activity in chemo-resistant EC mouse xenografts [344], suggesting its potential role as an important maintenance therapy to standard chemotherapy for patients with chemo-resistant EC.

Pioglitazone, a non-oncological drug, is a peroxisome proliferator-activated receptor gamma (PPAR-γ) agonist. Compared to the standard paclitaxel treatment, it showed significant dose-dependent anticancer activity against EC induced by N-ethyl-N-nitrosourea (ENU) and estradiol hexadrobenzoate (EHB) [345].

Gestrinone, initially designed as a contraceptive, now is being used as a therapy for endometriosis in clinical. According to a recent study, it has the potential to protect against gynecological cancers through regulation of the JNK-P21 axis, repurposing it may be beneficial to patients with gynecological cancers, especially for CC patients [346].

Perphenazine, approved for psychosis therapy, has been identified as a promising drug against both progesterone-sensitive and progesterone-resistant EC [347]. In addition, another antipsychotics drug, chlorpromazine (CPZ), showed a significant antitumor effect via upregulating the expression of progesterone receptor B (PRB) to sensitize progestin-resistant EC cells to MPA, which suggests that CPZ might be a potential candidate drug for conservative treatment for EC, and the combination of CPZ and MPA could act as a therapeutic option for progestin resistant EC patients [348].

These repurposing strategies have provided significant advancements in the treatment for gynecological cancers; however, most data are based on observational level. Therefore, long-term clinical trials are strongly deserved to further investigate the clinical benefits.

## Future perspectives: challenges and opportunities

Currently, nonsurgical treatments are urgently needed for gynecological malignancies, especially in endometrial cancer, for there are more young patients with a desire to maintain fertility due to the increasing incidence of EC and the younger age of onset [189, 349–351]. Therefore, acquired progesterone resistance should be thoroughly studied to provide therapeutic and prognostic targets or combination therapy strategies to overcome hormone therapy resistance in EC. Fortunately, antipsychotic drugs, perphenazine and CPZ, have been identified as promising drug candidates against progesterone-resistant EC [347, 348]. Previous study demonstrated that CPZ could upregulate the expression of PRB, suggesting that it might be a therapeutic option for progestin-resistant EC patients and act as a promising candidate drug for fertility-preserving options when combined with MPA [348]. Therefore, the potential clinical benefits should be further investigated by long-term clinical trials.

During the past decade, based on a further understanding of epigenetics, more epigenetic regulators with promising drug candidates have been validated in gynecological cancers, especially in EC and OC [38, 352]. A phase I study showed a synergetic effect of the combination of entionstat, a HDACi, and MPA in EC patients [93], so a treatment trial should be carried to further investigate the efficacy and toxicity of this combination therapy. Base on the knowledge that DNA methylation inhibitors may exert an effective role for EC treatment, so clinical trials involving these molecules are warranted to wait. Clinical trials of epigenetic monotherapy have proved disappointing in the treatment for OC patients [38, 101], suggesting that preclinical research should be focused on combination strategy with various epigenetic medications.

Although PARPis have made remarkable progress in the treatment of patients with EOC [353, 354], the application is restricted by the primary and acquired resistance. Several mechanisms underlying PARP inhibition resistance in OC have been reported [355–358], and new combinations of PARPis with antiangiogenic agents or immunotherapies are currently undergoing clinical evaluation [359–361], so more emphasis should be shifted to improve clinically applied strategies based on the results of these trials.

Furthermore, immunotherapies, mainly PD-1/PD-L1 inhibitors, have led to significant breakthroughs in personalized treatment for patients with dMMR or MSI EC and recurrent or metastatic CC [362]. However, only 10–30% of OC patients show long-term and durable responses to ICIs targeting PD-1/PD-L1 or CTLA-4, and the remaining majority do not respond [38]. Accordingly, how to tackle acquired resistance and the lack of response to ICIs is critical, and biomarkers, such as other immune checkpoints or coinhibitory receptors are mandatory to be identified. Notably, CD47 has become an immunotherapeutic hotspot and provided insights into new treatment options for patients with OC, based on the promising activities in preclinical models [303], thus related ongoing clinical trial (NCT03558139) are warranted to wait, and additional efforts should be made to facilitate the early application of research results to clinics.

## Conclusion

Significant breakthroughs have been made in the further understanding and improvement in treatment strategies for gynecological cancers, with a growing number of preclinical research and clinical trials on potential molecular drugs and their corresponding targets are being studied. Hormone receptor-targeted therapies, the signaling pathway molecules (e.g., PI3K/AKT/mTOR), immune-targeted strategies (e.g., anti-PD-1/ PD-L1 agents) might be the promising treatment for EC patients, based on the histological type and molecular subtypes. As for OC, PARPis have revolutionized the management of patients with EOC, with the recommendation of niraparib, rucaparib, and olaparib for maintenance therapy depending on the identification of HRD (e.g., g*BRCA*m). Immunotherapeutic strategies are expected to be clinically beneficial for CC patients, due to their persistent oncogenic HPV infection. However, challenges still exist for the conservative treatment for EC, improvement of immunotherapy response, and how to overcome acquired resistance of PARPis and ICIs. Therefore, numerous efforts should be made to deepen our understanding of pathogenesis driving gynecological malignancies, so as to revolutionize targeted therapy by exploring new specific biomarkers. Drug repositioning strategy should not be ignored, which has emerged as a promising antitumor treatment. In addition, considerable progresses are being made toward combination therapy in clinical research, so the long-term phase II–III clinical trials involving hotspot drug candidates that we discussed above are strongly warranted to wait.
